# Fluorine Effect in the Gelation Ability of Low Molecular Weight Gelators

**DOI:** 10.3390/gels8020098

**Published:** 2022-02-08

**Authors:** Paolo Ravarino, Nadia Di Domenico, Marianna Barbalinardo, Davide Faccio, Giuseppe Falini, Demetra Giuri, Claudia Tomasini

**Affiliations:** 1 Dipartimento di Chimica Giacomo Ciamician, Università di Bologna, Via Selmi, 2, 40126 Bologna, Italy; paolo.ravarino2@unibo.it (P.R.); nadia.didomenico@studio.unibo.it (N.D.D.); davide.faccio2@unibo.it (D.F.); giuseppe.falini@unibo.it (G.F.); 2 Istituto per lo Studio dei Materiali Nanostrutturati, Consiglio Nazionale delle Ricerche, (ISMN-CNR)-Via P. Gobetti 101, 40129 Bologna, Italy; marianna.barbalinardo@ismn.cnr.it

**Keywords:** fibers, fluorine atom, gelator, supramolecular gel, thixotropy, transparency

## Abstract

The three gelators presented in this work (Boc-D-Phe-L-Oxd-OH **F0**, Boc-D-F_1_Phe-L-Oxd-OH **F1** and Boc-D-F_2_Phe-L-Oxd-OH **F2**) share the same scaffold and differ in the number of fluorine atoms linked to the aromatic ring of phenylalanine. They have been applied to the preparation of gels in 0.5% or 1.0% *w*/*v* concentration, using three methodologies: solvent switch, pH change and calcium ions addition. The general trend is an increased tendency to form structured materials from **F0** to **F1** and **F2**. This property ends up in the formation of stronger materials when fluorine atoms are present. Some samples, generally formed by **F1** or **F2** in 0.5% *w*/*v* concentration, show high transparency but low mechanical properties. Two gels, both containing fluorine atoms, show increased stiffness coupled with high transparency. The biocompatibility of the gelators was assessed exposing them to fibroblast cells and demonstrated that **F1** and **F2** are not toxic to cells even in high concentration, while **F0** is not toxic to cells only in a low concentration. In conclusion, the presence of even only one fluorine atom improves all the gelators properties: the gelation ability of the compound, the rheological properties and the transparency of the final materials and the gelator biocompatibility.

## 1. Introduction

Low-molecular-weight (LMW) gelators are small molecules able to form supramolecular gels [1,2,3,4]. These are solid-like materials that can support their own weight when subjected to gravity. Among all the different types of gels, LMW gelators can form physical gels, consisting in a bundle of fibers formed through weak interactions that entrap the solvent. Particular attention should be paid to the functional groups present on these gelators, as aromatic rings, proton donors and acceptors, and hydrophobic moieties, that are particularly able to form these interactions [5,6,7,8,9].

It is possible to design new LMW gelators, following few guidelines. Among them, the insertion of moieties able to form weak bonds such as H-bonds and hydrophobic interactions is a common strategy. However, predicting if a molecule can form a gel is not straightforward. Among all the possible weak interactions, π–π stacking plays a pivotal role in governing the gelation process [10,11]. As halogen atoms usually enhance π interactions and allow the formation of additional weak bonds [12,13,14], their insertion on aromatic rings is another strategy to improve the gelling ability of a molecule.

Fluorine atom, in particular, has unique properties, such as high electronegativity, small size and low polarizability. The introduction of this halogen in peptide chains can affect the noncovalent interactions responsible for the self-assembly process. The C-H**^…^**F interactions were proved to be of the hydrogen bond type, resembling the C-H**^…^**N and C-H**^…^**O interactions and providing topologically similar supramolecular synthons [15].

The presence of a fluorine atom may significantly enhance weak interactions leading to important differences in the gelation abilities of LMW gelators. On the other hand, the gelation ability is also highly influenced by properties such as solubility and polarity [16,17,18]. Slight variations on the polarity of the gelator may either improve or reduce its gelling ability depending on the solvent used for the process [14,19,20]. Several works reported the influence of fluorine on the physical properties and self-assembly ability of different types of compounds [21,22,23,24]. Nilsson reported the efficient hydrogelation of Fmoc-protected pentafluorophenylalanine (Fmoc-F_5_-Phe) [25]. He also demonstrated an overall improvement on the gelation ability of Fmoc-Phe with the introduction of a fluorine atom in the para position (Fmoc-4F-Phe) [26]. In contrast, Metrangolo et al. demonstrated that the Fmoc-4-F-Phe resulted in certain conditions in an inefficient gelator compared to the other halogenated and nonhalogenated analogues [14].

We recently reported the gelation ability of protected and derivatized 3,4-difluorophenylalanine (F2-Phe) [27]. We compared the gelation ability of Boc-D-F_2_-Phe-OH (Boc = *tert*-butoxycarbonyl) with that of dipeptides where it was coupled with L-Oxd or D-Oxd (Oxd = 4-methyl-5-carboxy-oxazolidin-2-one) and we found out that Boc-D-F_2_Phe-L-Oxd-OH has the best performance among them. We used the Oxd moiety because we could demonstrate that its introduction in a peptide sequence strongly enhances the tendency of the molecule to self-assemble to form supramolecular materials [28,29,30,31,32,33,34,35,36].

Since the influence of fluorine on the formation of supramolecular gels is still controversial, in this paper, we show a systematic work where we analyzed and compared the effect of one or two fluorine atoms linked to the aromatic ring of a phenylalanine moiety, demonstrating that, in our case, these additional interactions enhance the properties of our gelators.

**Figure 1:**
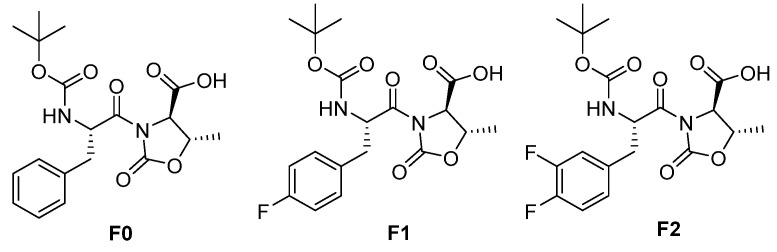
Chemical structure of the three gelators.

To carry on this study, we compared the gelation ability of three gelators: Boc-D-Phe-L-Oxd-OH, [37] Boc-D-F_1_Phe-L-Oxd-OH and Boc-D-F_2_Phe-L-Oxd-OH [27] (Phe= phenylalanine; F_1_Phe = 4-fluorophenylalanine; F_2_Phe = 3,4-difluorophenylalanine) (Figure 1). We report the preparation of gels starting from these three compounds and using several methodologies and triggers: solvent switch (from either ethanol or 2-propanol to water), pH change and calcium ions addition [38,39,40]. From the comparison of the outcomes, we demonstrate that the presence of even only one fluorine atom improves not only the gelation ability of the compound, but also the rheological properties of the final materials.

## 2. Results and Discussion

To carry out the analysis of the fluorine effect, we prepared the three gelators **F0**, 42 **F1** and **F2** 27 all sharing the Boc-L-Phe-D-Oxd-OH scaffold and differing for the number of fluorine atoms on the aromatic ring.

The heterocycle D-Oxd was prepared starting from D-threonine, according to the procedure reported by Falb [41], followed by esterification with benzyl bromide. Then three molecules **F0**, **F1** and **F2** were prepared in multigram scale by peptide coupling between the commercially available Boc-L-F_n_Phe-OH (*n* = 0, 1, 2) with D-Oxd-OBn. Finally, to obtain the free carboxy groups, the benzyl esters were removed by hydrogenolysis (Scheme S1, for the details see Supporting Information).

To understand if the fluorine effect may affect the gelling activity of the three gelators **F0**, **F1** and **F2** regardless the trigger, we compared their behavior using different triggers: solvent switch, pH changes and addition of calcium ions.

The three gelators were always tested both in 0.5% and 1.0% *w*/*v* concentration.

For the first trigger, we chose two different solvents (ethanol or 2-propanol) and water as antisolvent. To find the best conditions, we analyzed several mixtures of water/ethanol and water/2-propanol. The gelators were always dissolved in ethanol or 2-propanol in the required quantity, then water was added during ultrasound sonication. The mixtures were left resting for 16 h (for the details see Table S1). In Table 1 the samples are listed.

**Figure 2:**
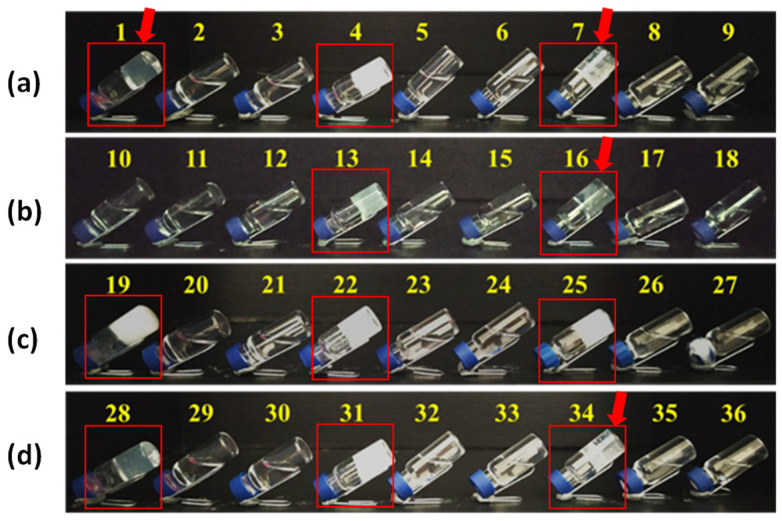
Picture of the samples formed with gelators **F0**, **F1** and **F2** and different mixtures of solvents: (**a**) gelators in 0.5% *w*/*v* concentration in mixtures of ethanol/water; (**b**) gelators in 0.5% *w*/*v* concentration in mixtures of 2-propanol/water; (**c**) gelators in 1.0% *w*/*v* concentration in mixtures of ethanol/water; (**d**) gelators in 1.0% *w*/*v* concentration in mixtures of 2-propanol/water. The red frames indicate the gel formed. The red arrows indicate the transparent gels according to the spectrophotometric analysis reported below.

In Figure 1, a photograph of the results is summarized and shows that the ratio between water and alcohol is crucial for the gel formation as only the mixtures ethanol/water and 2-propanol/water in 3:7 ratio successfully furnish a gel. In Figure 2, the gels formed (**1**, **4**, **7**, **13**, **16**, **19**, **22**, **25**, **28**, **31** and **34**) are highlight by a red frame.

Then, the gelation ability of **F0**, **F1** and **F2** in PB (phosphate buffer) solution was tested, using as a trigger both the pH change method [39] and the addition of calcium chloride [43] (Table 2). The use of PB solution as a solvent in 0.03 or 0.06 M concentration, according to the gelator concentration, was required to avoid a high pH that favors the hydrolysis of gelators **F1** and **F2**, as we reported in our previous paper (for details see Table S2) [27].

In both cases, the samples were dissolved in phosphate buffer solution at pH 7.4 (see section Materials and Methods for more details), as in pure water the molecules are not soluble. In examples **37**–**39** (**F0**, **F1** and **F2**, all in 0.5% *w*/*v* concentration) and **43**–**45** (**F0**, **F1** and **F2**, all in 1.0% *w*/*v* concentration), the solution 1.4 equivalents of GdL (glucono-δ-lactone) were added. Its slow hydrolysis reduces the pH value leading to the protonation of the carboxylic moiety and to the formation of the fibrils that end up in the hydrogel formation.

In examples **40**–**42** (**F0**, **F1** and **F2**, all in 0.5% *w*/*v* concentration) and **46**–**48** (**F0**, **F1** and **F2**, all in 1.0% *w*/*v* concentration), a 0.06 M aqueous solution of CaCl_2_ (1.0 equivalents) has been added to the solutions of gelators in PBS. In this case, the crosslinking between two carboxylic moieties leads to the formation of fibrils that trap the solvent to obtain the hydrogel.

**Figure 3:**
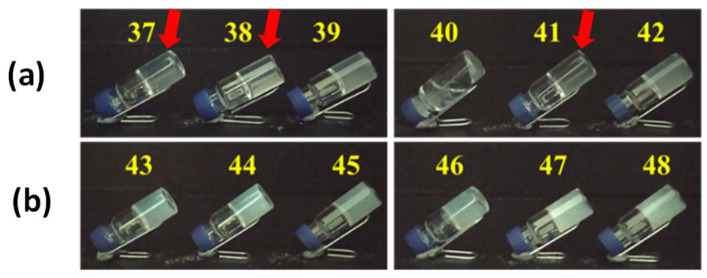
Picture of the samples formed with gelators **F0**, **F1** and **F2** in PBS with different triggers: (**a**) gelators in 0.5% *w*/*v* concentration with GdL (**37**–**39**) and CaCl_2_ (**40**–**42**) in PBS; (**b**) gelators 1.0% *w*/*v* concentration with GdL (**43**–**45**) and CaCl_2_ (**46**–**48**) in PBS. The red arrows indicate the transparent gels according to the spectrophotometric analysis (see Table 3).

The results obtained for the three gelators are reported in Figure 3: only sample **40** containing molecule **F0** is not a gel. It is interesting to notice that several gels (mainly in 0.5% *w*/*v* concentration) appear very transparent.

To confirm the transparency of the gels, we repeated their preparation in cuvettes to check their absorbance by spectrophotometer analysis in the visible range between 400 and 700 nm, and we accepted as transparent the sample that has a transmittance ≥50%, measured at 630 nm (Table 3). The value at 630 nm was chosen as a middle value in the range 560–700 nm, where the molecules showed the highest transparency (a decrease in absorption). The seven transparent samples (**1**, **7**, **16**, **34**, **37**, **38** and **41**) are shown in Figure 2 and Figure 3 with a red arrow. In Figure S1 the absorbance spectra of all the gels are reported.

**Figure 4:**
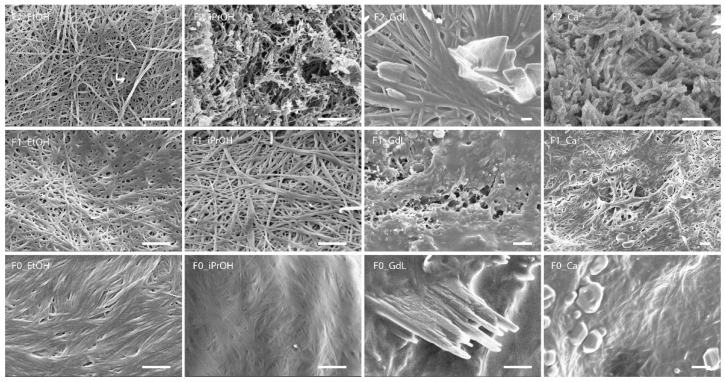
SEM image of the xerogel of samples. The samples **19**, **22** and **25** are indicated as F0_EtOH, F1_EtOH and F2_EtOH, respectively. The samples **28**, **31** and **34** are indicated as F0_iPrOH, F1_ iPrOH and F2_ iPrOH, respectively. The samples **43**, **44** and **45** are indicated as F0_GdL, F1_ GdL, and F2_ GdL respectively. The samples **46**, **47** and **48** are indicated as F0_Ca^2+^, F1_ Ca^2+^ and F2_ Ca^2+^, respectively. Scale bar: 1 μm.

A further characterization was obtained by the analysis of the structure of the xerogel by optical microscope (OM) and scanning electron microscope (SEM) to check if differences may be found in the fibers networks. The morphology of the dried hydrogels was analyzed through an optical microscope and all the images are shown in Figures S2–S5. In the largest part of the xerogels, the pictures show that complex networks of fibers are present in almost all the materials and this effect is more evident in the higher magnification 20×. A further confirmation of these results has been obtained with the analysis of the SEM images of the xerogels in 1.0% *w*/*v* concentration (Figure 4, Figures S6 and S7). In general, fibers are more visible for xerogels prepared with **F1** and **F2,** while in the samples prepared with **F0** the fibers are hardly visible. The SEM images also show an effect of the solvent on the organization of the molecules at the superstructure level. In ethanol and 2-propanol, a similar behavior is observed among the different molecules. All have the tendency to assembly in fibers. This effect is much more marked for the **F1** and **F2** molecules, the **F0** xerogel molecule appears as an amorphous aggregate in which some fibers are dispersed. When xerogels are obtained from chemical systems containing GdL or calcium ions, the formation of the fibers is less evident with respect to xerogels from EtOH and iPrOH, although the greater aptitude of **F1** and **F2** to form fibers is confirmed. In these last xerogels, the presence of crystalline mineral deposits is also observed.

The SEM considerations on molecules assembly to form superstructures are supported by the X-ray powder diffraction data (Figures S8–S10). They show the presence of crystalline peaks, and thus of an ordered assembly, for the xerogels obtained from **F1** and **F2** in ethanol or 2-propanol. While from the same solvents **F0** xerogels produce mainly an amorphous band. The diffraction patterns from the xerogels obtained from chemical systems containing GdL or Ca^2+^ show weak diffraction peaks from the molecules together with diffraction peaks from the mineral phases.

The analysis of the samples was pursued with the study of the mechanical properties of the gels. New gels samples were prepared in a 2 mL Sterilin Cups^®^ to check their stiffness and thixotropic attitude. The complete series of the amplitude measurements, that have been measured in triplicate, are reported in Figures S11 and S12. In general, they show a high variability of G’ with values ranging from 10^3^ to 10^6^.

The analysis of the time sweeps of gels formed with the pH variation methods is reported in Figure S13 and was performed to check the time required to form the gels with a slow modification of the pH of the solution. For all the gels prepared in 0.5% *w*/*v* concentration, the complete formation of the gels was complete within 4 h, while the time required to form the gels in 1.0% *w*/*v* concentration is 2 h.

**Figure 5:**
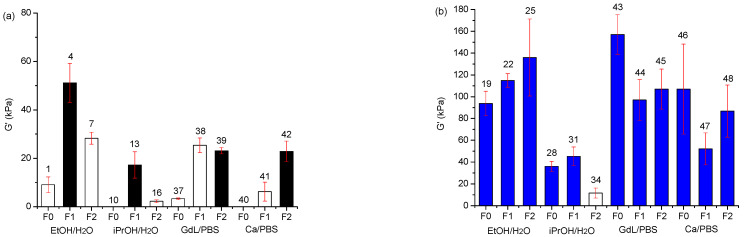
Summary of the stiffness of the gels (γ = 0.068%) by means of G’ (kPa). (**a**) Gels in 0.5 *w*/*v* concentration; (**b**) gels in 1% *w*/*v* concentration. The empty bars are referred to transparent gels with transmittance T ≥ 50%, solid bars are instead referred to gels with T ≤ 50%. Error bars are reported with red lines. Samples **10** and **40** did not form a gel.

To have a better look at these results and to correlate the strength with the transparency of the gels, we summarized all the G’ values of the samples in Figure 5a,b, showing the transparent samples with empty bars and the opaque samples with solid bars. The optical and mechanical properties of the gels are reported in Table S3.

As we could foresee, the transparent gels are generally quite weak, as their G’ values never overcome 10 kPa. We have only two exceptions that are samples **7** (**F2** in ethanol/water in 0.5% *w*/*v* concentration) and **38** (**F1** in aqueous PBS/GdL in 0.5% *w*/*v* concentration), that show good mechanical properties coupled with high transparency.

**Figure 6:**
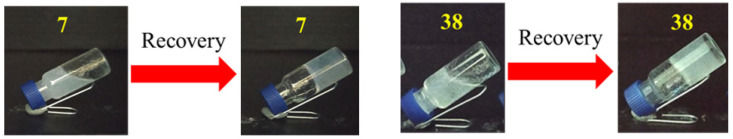
Thixotropic behavior of gels **7** and **38** formed after vigorous shaking (**left**) and 16 h of recovery (**right**).

Finally, the thixotropy of the samples was analyzed both with the rheological analysis (Figures S14 and S15) and by breaking them with vigorous shaking and overnight recovery (16 h) (Figure 6 and Figure S16). While the analysis with the rheometer demonstrates that all the samples can form again, the more aggressive physical shaking shows that samples **13**, **16**, **41** and **42** do not recover in 16 h. We could notice that the strong and transparent gels **7** and **38** have excellent thixotropic properties both with the rheometer analysis and after vigorous shaking (Figure 6).

Another important property that must be assessed for the application of gelators **F0**, **F1** and **F2** to the formation of supramolecular materials, is their biocompatibility.

In a recent paper, we demonstrated the biocompatibility of other gelators derived from the Oxd moiety [30]. Molecules **F0**, **F1** and **F2** were dissolved in ethanol, then the solutions were diluted in Dulbecco’s phosphate-buffered saline (DPBS), with final concentrations of 5 mg/mL and 0.5 mg /mL. Then the solutions were administered to mouse embryonic fibroblast (NIH-3T3).

**Figure 7:**
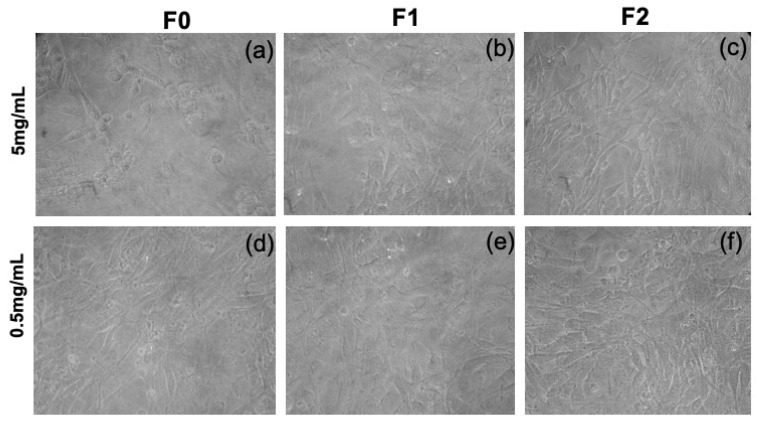
Optical micrographs of cells after 24 h (**a**) **F0**, (**b**) **F1**, (**c**) **F2** at concentration 5 mg/mL, (**d**) **F0** (**e**) **F1** and (**f**) **F2** at concentration 0.5 mg/mL.

Fibroblast cells were exposed to **F0, F1** and **F2** for 24 h, then their mitochondrial function was measured by the MTT viability essay. A significant decrease of this function was detected after exposure to **F0** at the highest concertation (5 mg/mL), as cell viability drops dramatically to 40% (Table 4). In contrast, the presence of **F1** and **F2** at the same concentration did not show a cytotoxic effect. Conversely, the 0.5 mg/mL concentration in **F0, F1** and **F2** does not reduce cell viability. Figure 7 shows the morphology of the fibroblasts in the presence of the three molecules. In all cases, the fibroblasts do not change their cell morphology compared to the control where only DPBS was administered (Figure S17). From these preliminary results, we can state that the gelators **F0, F1** and **F2** are not toxic to cells in low concentration, and that **F1** and **F2** are not toxic to cells even in high concentrations.

## 3. Conclusions

In this manuscript we reported a comprehensive and systematic study on three gelators that share the same scaffold Boc-D-F_n_Phe-L-Oxd-OH (*n* = 0, 1, 2) and differ for the number of fluorine atoms linked to the aromatic ring. In our opinion, this study was necessary to shed light on the effect of fluorine atoms linked to molecules that act as gelators, as the influence of fluorine on the formation of supramolecular gels is still controversial. The three molecules Boc-D-Phe-L-Oxd-OH, Boc-D-F_1_Phe-L-Oxd-OH and Boc-D-F_2_Phe-L-Oxd-OH have been applied to the preparation of gels in 0.5% or 1.0% *w*/*v* concentration, using three methodologies: solvent switch method (from either ethanol or 2-propanol to water), pH change method and calcium ions addition. From the comparison of the outcomes, the general trend is an increase in tendency to form structured materials from **F0** to **F1** to **F2**, as shown by the microscope analysis. This property ends up into the formation of stronger materials that contain fluorine atoms. The transparency of the materials is present in some samples, generally formed by **F1** or **F2** in 0.5% *w*/*v* concentration, but in most cases the samples are quite weak. We could find two cases (sample **7**: **F2** in 0.5% *w*/*v* concentration in EtOH/H_2_O 7:3 and sample **38**: **F1** in 0.5% *w*/*v* concentration in PBS/GdL) that show good mechanical properties coupled with high transparency.

The biocompatibility of the gelators was assessed exposing them to fibroblast cells. Gelators **F0, F1** and **F2** are not toxic to cells in low concentration, while **F1** and **F2** are not toxic to cells even in high concentration. The biocompatibility of the compounds, coupled with the high recovery properties and transparency in the visible region of the gels make these materials good candidates for biological and biomedical applications, such as cell culture and drug delivery.

In conclusion, we demonstrate that the presence of even only one fluorine atom improves all the gelators properties: the gelation ability of the compound, the rheological properties and the transparency of the final materials and the gelator biocompatibility.

## 4. Materials and Methods

### 4.1. General Remarks for the Synthetic Procedure

Solvents were dried by distillation before use. All reactions were carried out in dried glassware. The melting points of the compounds were determined in open capillaries and are uncorrected. All compounds were dried in vacuo and all the sample preparations were performed in a nitrogen atmosphere.

High-quality infrared spectra (64 scans) were obtained at 2 cm^−1^ resolution with an ATR-IR Agilent (Santa Clara, CA, USA) Cary 630 FTIR spectrometer. NMR spectra were recorded with a Varian (Palo Al-to, CA, USA) Inova 400 spectrometer at 400 MHz (^1^H NMR), at 100 MHz (^13^C NMR) and at 376.5 MHz (^19^F NMR). Chemical shifts are reported in δ values relative to the solvent peak. HPLC-MS Agilent (Santa Clara, CA, USA) equipped with a column C18 was used to check the purity of compounds. A Jasco (Mary’s Court, MD, USA) P-2000 Polarimeter was used to check the optical rotatory power of the compounds.

### 4.2. Synthesis of F0, F1, and F2

H-L-Oxd-OBn was prepared according to the procedure reported in reference [9]. Synthesis of compounds **F0**, **F1** and **F2** was carried out following the same procedure. Boc-D-F_n_-Phe-OH (with *n* = 0, 1 or 2) (2.00 mmol) was dissolved in 20 mL of acetonitrile (10 mL) and HBTU (2.20 mmol) was added. The mixture was stirred at room temperature for 10 min. A solution containing H-L-Oxd-OBn (2.00 mmol) and DIEA (4.40 mmol) in acetonitrile (10 mL) was then added dropwise to the first one. The mixture was stirred for 2 h, then the solvent was removed under reduced pressure and replaced with ethyl acetate (40 mL). The organic mixture was washed with H_2_O (10 mL), 1M aqueous HCl (10 mL) and brine (10 mL), then it was dried over Na_2_SO_4_ and the solvent evaporated under vacuum. The solid obtained was finally purified through flash chromatography (dichloromethane:ethyl acetate 95:5). All the Boc-D-F_n_-Phe-L-Oxd-OBn samples were obtained as white solids and directly hydrogenolysed. They were dissolved in methanol to obtain a concentration of 10 mg/mL, then the 10% *w*/*w* of Pd/C was added. The reaction mixture was vigorously stirred under hydrogen atmosphere for 2 h at room temperature, then it was filtered over a celite pad. The solvent was removed under reduced pressure and the product was used without further purification. Characterization of **F0** matched the values reported in reference [10], characterization of **F1** is reported in the Supplementary File, and characterization of **F2** matched the values reported in reference [7].

### 4.3. Gel Preparation

The gels used for absorbance measurements were directly prepared in plastic cuvettes, the gels used for pictures were prepared in a 1.5 mL vial and the gels used for the rheological analysis were prepared in Sterilin Cups^®^. All the gels were left to rest for 16 h at room temperature before their use.

For the gels **1–36,** the gelators were dissolved in the organic solvent (ethanol or 2-isopranol) by alternating manual shaking and ultrasound sonication, then water was added during sonication.

The gels **37**–**48** were all dissolved using a phosphate buffer (PB) solution whose final concentration is 0.03 M. PB solution was prepared dissolving KH_2_PO_4_ in water and adjusting the pH to a final value of 7.4 by adding NaOH 1 M. For the gels **37–39,** the gelators were dissolved in a 0.03 M PB solution at pH 7.4, then 1.4 eq of solid GdL was added. Samples **40**–**42** were prepared by dissolving the gelators in a 0.04 M PB solution at pH 7.4, then 1.0 eq of 0.06 M CaCl_2_ aqueous solution was added. Samples **43**–**45** were prepared following the same procedure used for samples **37**–**39**, using a 0.06 M PB solution. Samples **46**–**48** were prepared following the same procedure used for samples **40**–**42**, using a 0.08 M PB solution.

### 4.4. Rheological Analysis

All rheological measurements were performed using an Anton Paar (Graz, Austria) MCR102 rheometer. A vane and cup measuring system was used, setting a gap of 2.1 mm. The gels were prepared as described and tested directly in the Sterilin Cup^®^ which fits in the rheometer. Time sweep experiments were performed at 23 °C (controlled by an integrated Peltier system) using a constant shear strain (γ) of 0.5% and a constant angular frequency (ω) of 10 rad/s, collecting 1 point every 20 s. Oscillatory amplitude sweep experiments (γ: 0.01–100%) were also performed at 23 °C using a constant angular frequency of 10 rad/s. Step strain experiments were performed on hydrogels, subjecting the sample to consecutive deformation and recovery steps. The recovery step was performed by keeping the sample at a constant strain γ = 0.03%, i.e., within the LVE region, for a period of 400 s. The deformation step was performed by applying to the gel a constant strain of γ = 100%, i.e., above the LVE region of the sample, for a period of 300 s. The cycles were performed at a fixed frequency of ω = 10 rad s−1 and repeated three times.

### 4.5. Optical Microscope Images

The optical microscope images were recorded using an ECLIPSE Ti2 Inverted Research Microscope with a 10× or 20× magnifier. A piece of the gel sample prepared in the Sterilin Cups^®^ was cut using a bistoury and analyzed after complete drying.

### 4.6. Scanning Electron Microscope Images

Scanning electron micrographs were recorded on carbon-coated samples using a Zeiss LEO 1530.

### 4.7. X-ray Powder Diffraction Analysis

X-ray powder diffraction (XRPD) measurements were performed with a PanAnalytical X’Pert Pro diffractometer equipped with X’Celerator detector with Cu Kα radiation. The samples were ground before the measurements.

### 4.8. Cell Viability Measurement

Mouse embryonic fibroblast (NIH-3T3) cells were cultured under standard conditions in the MEM medium, supplemented with 10% (*v*/*v*) FBS, 2 mM L-glutamine, 0.1 mM MEM Non-Essential Amino Acids (NEAA), 100 U mL^−1^ penicillin and 100 U mL^−1^ streptomycin. Cells were seeded in 96-well plates at a density 10^5^ cells per cm^2^ and grown for 24 h before exposure to **F0**, **F1** and **F2**. Cells were incubated for 24 h in a humidified incubator set at 37 °C [44]. Cellular viability was assessed by MTT assay, measuring intracellular reduction of tetrazolium salts into purple formazan by viable cells [45]. Cells were incubated with MTT solution (5 mg mL^−1^ MTT) for 2 h at 37 °C, 5% CO_2_. Subsequently, the MTT solution was discarded and dimethyl sulfoxide (DMSO) was added to each well. Optical density (OD) was read on a microplate reader at 550 nm (Thermo Scientific Varioskan Flash Multimode Reader). Cell viability for each treatment was calculated as the ratio of the mean OD of replicated wells relative to that of the control. All data represented the mean ± standard deviation.

### 4.9. Spectrophotometric Analysis

The gel samples were directly prepared into disposable cuvettes with 10 mm optical path. The spectrophotometric analyses were performed using a Cary 300 UV-vis double beam spectrophotometer, using a cuvette with the solvent as reference.

## Figures and Tables

**Table 1:** List of the samples prepared with the solvent switch method.

Gelator	Solvent	Trigger	Conc. (*w*/*v*)	Sample	Conc. (*w*/*v*)	Sample
**F0**	EtOH (30%)	Water (70%)	0.5%	1	1.0%	19
	EtOH (50%)	Water (50%)	0.5%	2	1.0%	20
	EtOH (70%)	Water (30%)	0.5%	3	1.0%	21
**F1**	EtOH (30%)	Water (70%)	0.5%	4	1.0%	22
	EtOH (50%)	Water (50%)	0.5%	5	1.0%	23
	EtOH (70%)	Water (30%)	0.5%	6	1.0%	24
**F2**	EtOH (30%)	Water (70%)	0.5%	7	1.0%	25
	EtOH (50%)	Water (50%)	0.5%	8	1.0%	26
	EtOH (70%)	Water (30%)	0.5%	9	1.0%	27
**F0**	^i^PrOH (30%)	Water (70%)	0.5%	10	1.0%	28
	^i^PrOH (50%)	Water (50%)	0.5%	11	1.0%	29
	^i^PrOH (70%)	Water (30%)	0.5%	12	1.0%	30
**F1**	^i^PrOH (30%)	Water (70%)	0.5%	13	1.0%	31
	^i^PrOH (50%)	Water (50%)	0.5%	14	1.0%	32
	^i^PrOH (70%)	Water (30%)	0.5%	15	1.0%	33
**F2**	^i^PrOH (30%)	Water (70%)	0.5%	16	1.0%	34
	^i^PrOH (50%)	Water (50%)	0.5%	17	1.0%	35
	^i^PrOH (70%)	Water (30%)	0.5%	18	1.0%	36

**Table 2:** List of the samples prepared with the pH change method and the addition of calcium chloride.

Gelator	Trigger (equiv.)	Conc. (*w*/*v*)	Sample	Conc. (*w*/*v*)	Sample
**F0**	GdL (1.4)	0.5%	37	1.0%	43
**F1**	GdL (1.4)	0.5%	38	1.0%	44
**F2**	GdL (1.4)	0.5%	39	1.0%	45
**F0**	CaCl_2_ (1.0)	0.5%	40	1.0%	46
**F1**	CaCl_2_ (1.0)	0.5%	41	1.0%	47
**F2**	CaCl_2_ (1.0)	0.5%	42	1.0%	48

**Table 3:** Analysis of the transparency of the gel samples taken in a cuvette. Transparent samples (with T ≥ 50%) are in bold.

Sample	A (λ = 630 nm)	T (%)	Sample	A (λ = 630 nm)	T (%)
**1**	**0.17469**	**66.88**	**37**	**0.24547**	**56.82**
4	1.96473	1.08	**38**	**0.19197**	**64.27**
**7**	**0.26185**	**54.72**	39	0.33574	46.16
19	1.26706	5.41	43	0.8128	15.39
22	2.98876	0.10	44	0.30614	49.41
25	2.04926	0.89	45	1.32719	4.71
13	2.83436	0.15	**41**	**0.12512**	**74.97**
**16**	**0.23566**	**58.12**	42	0.66275	21.74
28	0.68608	20.60	46	1.86026	1.38
31	2.92284	0.12	47	0.93284	11.67
**34**	**0.11273**	**77.14**	48	1.99850	1.00

**Table 4:** Cell viability of NIH-3T3 cells after 24 h of treatment with 5 mg/mL and 0.5 mg/mL of **F0, F1** and **F2**.

Samples	Cell Viability (%) (Control)	Cell Viability (%) (5 mg/mL)	Cell Viability (%) (0.5 mg/mL)
DPBS	100 ± 7	-	-
**F0**	-	40 ± 5	89 ± 5
**F1**	-	77 ± 7	90 ± 9
**F2**	-	73 ± 9	99 ± 4

## Supplementary Materials

The following supporting information can be downloaded at: https://www.mdpi.com/article/10.3390/gels8020098/s1, Scheme S1. Reagents and conditions for the preparation of **F0, F1** and **F2**; Characterization of compounds **F0, F1** and **F2**; NMR and IR spectra of the compound **F1**; Table S1. Detailed list of the samples prepared with the solvent switch method; Table S2. Detailed list of the samples prepared with the pH change method and the addition of calcium chloride; Figure S1. Absorbance spectra of the gels samples; Figure S2. Morphology of the dried hydrogels **1**, **4**, **7**, **13** and **16**, analyzed through an optical microscope with different magnifications; Figure S3. Morphology of the dried hydrogels **19**, **22**, **25**, **28**, **31** and **34**, analyzed through an optical microscope with different magnifications; Figure S4. Morphology of the dried hydrogels **37**, **38**, **39**, **41** and **42**, analyzed through an optical microscope with different magnifications; Figure S5. Morphology of the dried hydrogels **43**, **44**, **45**, **46**, **47** and **48**, analyzed through an optical microscope with different magnifications; Figures S6 and S7. SEM images of the dried hydrogels **19**, **22**, **25**, **28**, **31**, **34** and **43**–**48**; Figures S8–S10. X-ray powder diffraction patterns of the xerogels from molecules **F0, F1** and **F2**; Figure S11. Amplitude sweep analysis of the samples **1**, **4**, **7**, **13**, **16**, **19**, **22**, **25**, **28**, **31** and **34**; Figure S12. Amplitude sweep analysis of the samples **37**–**39** and **41**–**48**; Figure S13. Time sweep analysis of the samples **37**–**39** and **43**–**45** obtained with the addition of GdL; Table S3. Summary of the properties of gels; Figure S14. Thixotropic behavior of the samples **1**, **4**, **7**, **13**, **16**, **19**, **22**, **25**, **28**, **31** and **34** analyzed with the rheometer; Figure S15. Thixotropic behavior of the samples 37, 38, 39 and 41–48, analyzed with the rheometer; Figure S16. Thixotropic behavior of the gels formed after vigorous shaking (left) and 16 h of recovery (right); Figure S17. Optical micrograph of cells in DPBS (control).
