# Supplementary material for: Fluorine Effect in the Gelation Ability of Low Molecular Weight Gelators
Source: Gels. 2022 Feb 8;8(2):98. doi: 10.3390/gels8020098 (PMC8871896; doi:10.3390/gels8020098)
Supplement: Supplementary file 1 [file gels-08-00098-s001.zip › gels-1557704-supplementary.pdf]

Supporting Information

Fluorine Effect in the Gelation Ability of Low Molecular Weight Gelators

Paolo Ravarino [1], Nadia Di Domenico [1], Marianna Barbalinardo [2], Davide Faccio [1], Giuseppe Falini [1], Demetra Giuri [1*], and Claudia Tomasini [1*]

[1] Dipartimento di Chimica "Giacomo Ciamician" Università di Bologna – Via Selmi, 2 – 40126 Bologna – Italy [2] Istituto per lo Studio dei Materiali Nanostrutturati, Consiglio Nazionale delle Ricerche, (ISMN-CNR) - Via P. Gobetti 101 – 40129 Bologna Italy - mail: claudia.tomasini@unibo.it

**Scheme S1.** Reagents and conditions for the preparation of **F0, F1** and **F2**	Page S2
Characterization of compounds **F0, F1** and **F2**	Page S3
NMR and IR spectra of the compound **F1**	Pages S4-S8
**Table S1.** Detailed list of the samples prepared with the solvent switch method	Page S9
**Table S2.** Detailed list of the samples prepared with the pH change method and the addition of calcium chloride	Page S10
**Figure S1.** Absorbance spectra of the gels samples	Page S11
**Figure S2.** Morphology of the dried hydrogels **1, 4, 7, 13** and **16,** analysed through an optical microscope with different magnifications	Page S12
**Figure S3.** Morphology of the dried hydrogels **19, 22, 25, 28, 31** and **34**, analysed through an optical microscope with different magnifications	Page S13
**Figure S4.** Morphology of the dried hydrogels **37, 38, 39, 41** and **42,** analysed through an optical microscope with different magnifications	Page S14
**Figure S5.** Morphology of the dried hydrogels **43, 44, 45, 46, 47** and **48,** analysed through an optical microscope with different magnifications	Page S15
**Figures S6-S7.** SEM images of the dried hydrogels **19, 22, 25, 28, 31, 34** and **43-48**	Pages S16-S17
**Figures S8-S10.** X-ray powder diffraction patterns of the xerogels from molecules **F0**, **F1** and **F2**	Pages S18-S19
**Figure S11.** Amplitude sweep analysis of the samples **1, 4, 7, 13, 16, 19, 22, 25, 28, 31** and **34**	Page S20
**Figure S12.** Amplitude sweep analysis of the samples **37, 38, 39** and **41-48**	Page S21
**Figure S13.** Time sweep analysis of the samples **37-39** and **43-45** obtained with the addition of GdL	Page S22
**Table S3.** Summary of the Properties of Gels	Page S23
**Figure S14.** Thixotropic behaviour of the samples **1, 4, 7, 13, 16, 19, 22, 25, 28, 31** and **34** analysed with the rheometer	Page S24
**Figure S15.** Thixotropic behaviour of the samples **37, 38, 39** and **41-48**, analysed with the rheometer	Page S25
**Figure S16.** Thixotropic behaviour of the gels formed after vigorous shaking (left) and 16 hours of recovery (right)	Page S26
**Figure S17.** Optical micrograph of cells in DPBS (control)	Page 27

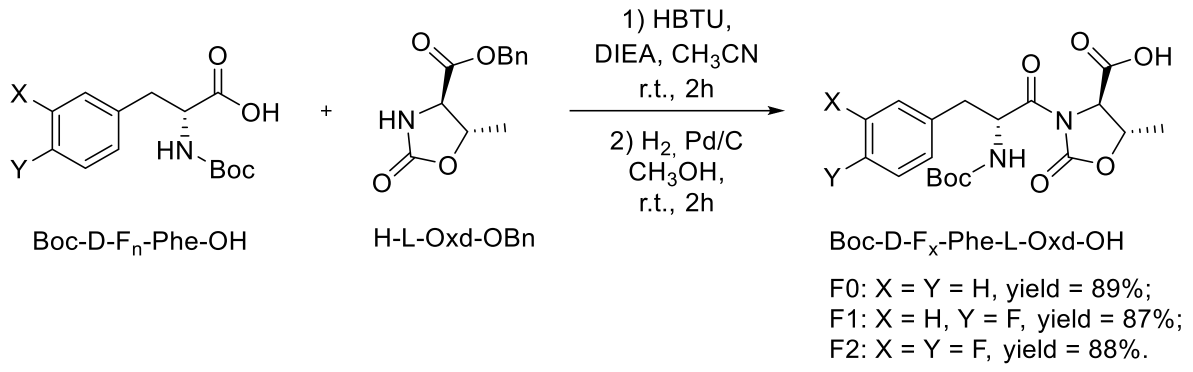

Scheme S1. Reagents and conditions: 1) Boc-D-F_n-Phe-OH (1.0 eq), H-L-Oxd-OBn (1.0 eq), HBTU (1.1 eq), DIEA (2.2 eq) in CH_3CN (concentration of the first two reagents is 0.1 M) for 2 h at r.t.. 2) Boc-D-F_n-Phe-L-Oxd-OBn (1.0 eq) and Pd/C (10% w/w) in CH_3OH (concentration of the first reagent is 10 mg/mL) for 2 h at r.t. under H_2 atmosphere.

Characterization of compounds F0, F1 and F2

Boc-D-Phe-L-Oxd-OH, **F0**

Characterisation matched literature values. See ref. [1].

Boc-D-F_1Phe-L-Oxd-OH, **F1**

The product **F1** is obtained as a white solid with an 87% yield. M.p. = 172-178°C; $[\alpha]_D^{25}$= -32.2° (c = 0.5 in CH_3OH); IR (ATR-IR): ν 3370, 3364, 2984, 2937, 1778, 1720, 1687, 1603, 1510 cm^{-1}; ^1H NMR (CD_3OD, 400 MHz): δ 1.31 (9H, s, CH_3 *t*-Bu), 1.47 (3H, d, J = 6.4 Hz, CH_3 Oxd), 2.71 (1H, dd, J = 10.0, 13.2 Hz, CH benzyl), 3.10 (1H, m, CH benzyl), 4.41 (1H, d, J = 3.6 Hz, $C_\alpha H$ Oxd), 4.69 (1H, m, $C_\beta H$ Oxd), 5.63 (1H, dd, J = 4.0, 9.6 Hz, $C_\alpha H$ F-Phe), 6.97 (2H, m, CH Ar), 7.30 (2H, m, CH Ar); ^{13}C (CD_3OD_3, 100 MHz): δ 19.77, 27.18, 37.19, 54.37, 61.92, 74.57, 79.04, 114.32, 114.53, 130.85, 130.93, 132.88, 152.28, 155.89, 163.07, 170.04, 172.44; ^{19}F NMR (CD_3OD, 376.5 MHz): δ -118.63, -118.46.

Boc-D-F_2Phe-L-Oxd-OH, **F0**

Characterisation matched literature values. See ref. [2].

References

1. Milli, L.; Castellucci, N.; Tomasini, C. Turning around the L-Phe-D-oxd moiety for a versatile low-molecular-weight gelator. *European J. Org. Chem.* **2014**, *2014*, 5954–5961, doi:10.1002/ejoc.201402787.

2. Ravarino, P.; Giuri, D.; Faccio, D.; Tomasini, C. Designing a transparent and fluorine containing hydrogel. *Gels* **2021**, *7*, 1–10, doi:10.3390/gels7020043.

^{1}H NMR spectrum of compound **F1**, acquired in CD_3OD

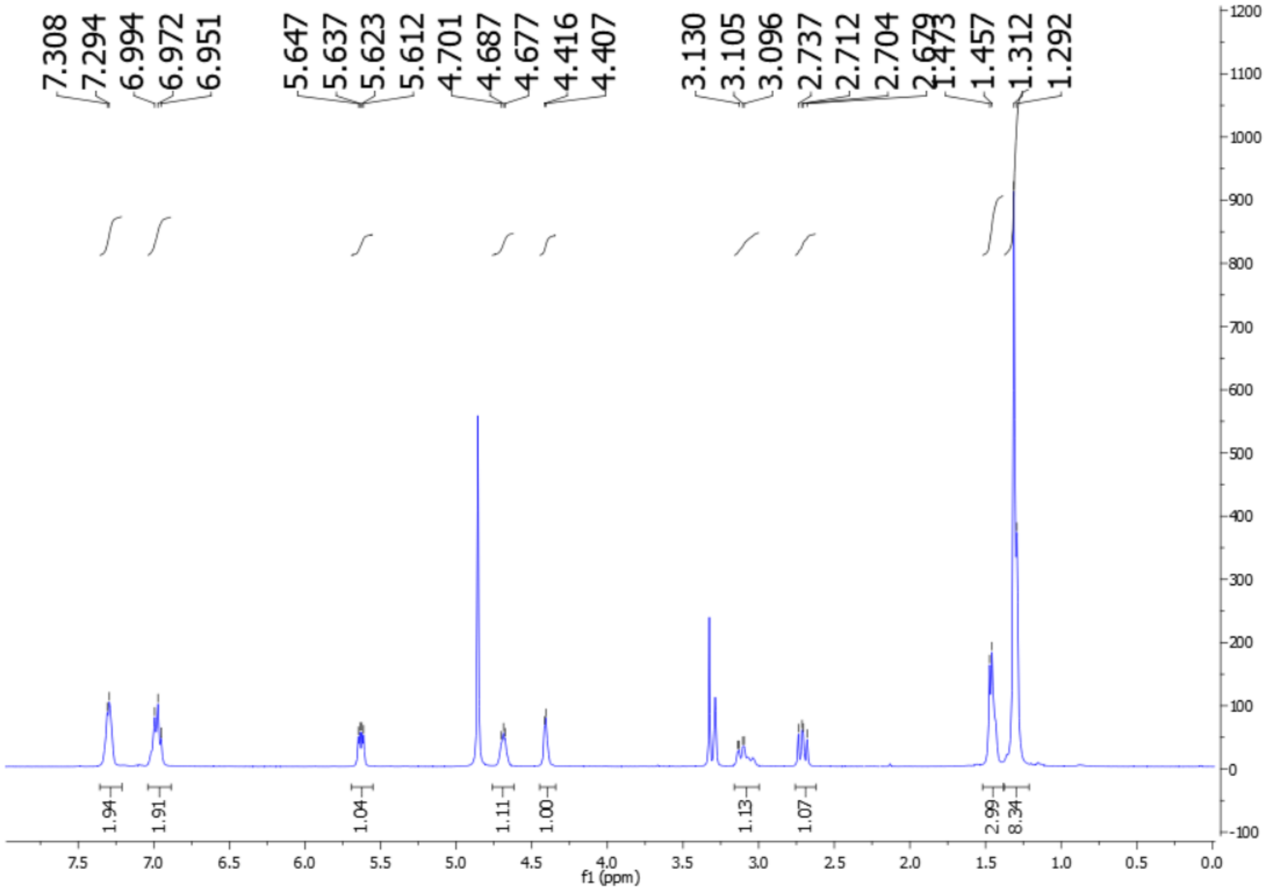

COSY NMR spectrum of compound **F1**, acquired in CD_3OD

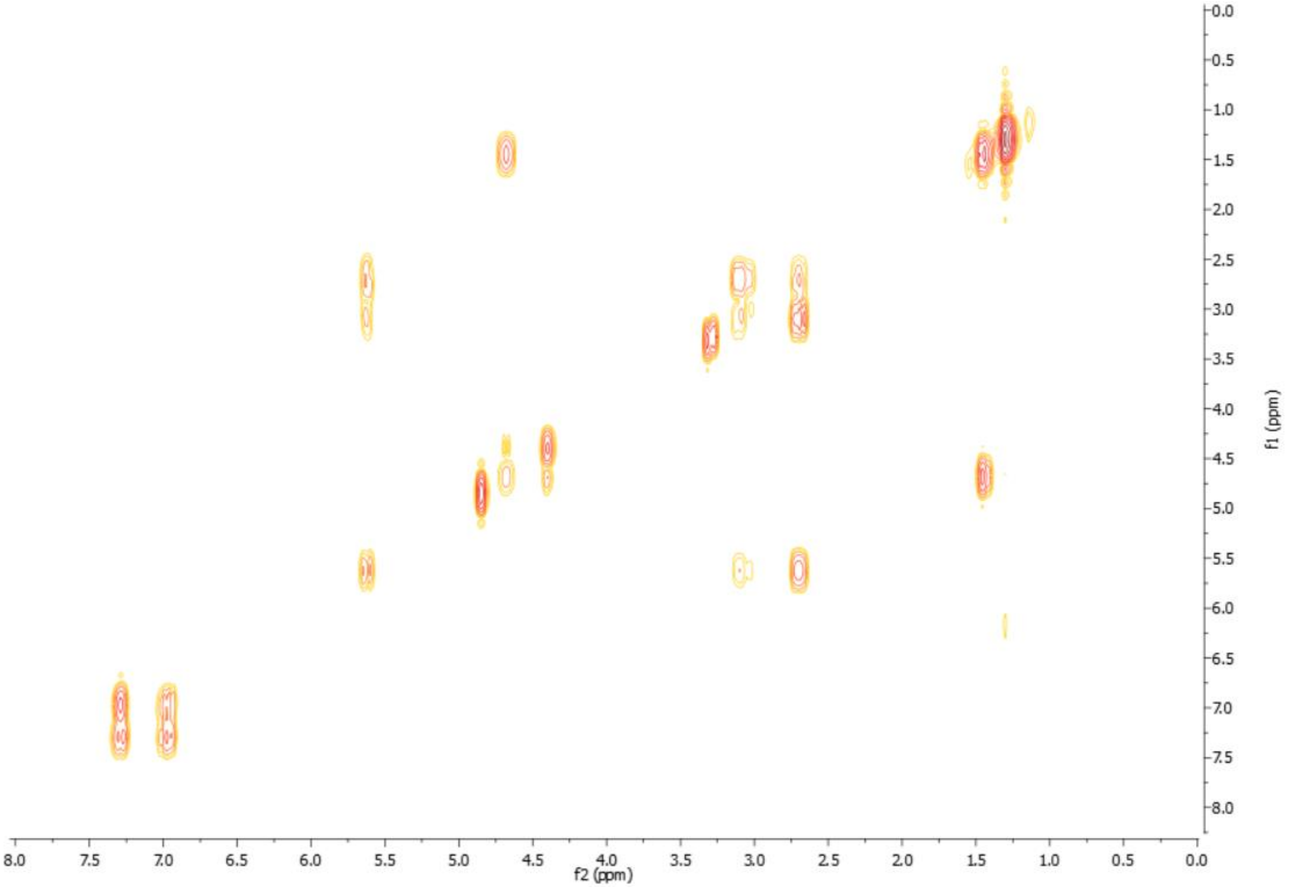

^{13}C NMR spectrum of compound F1, acquired in CD_3OD.

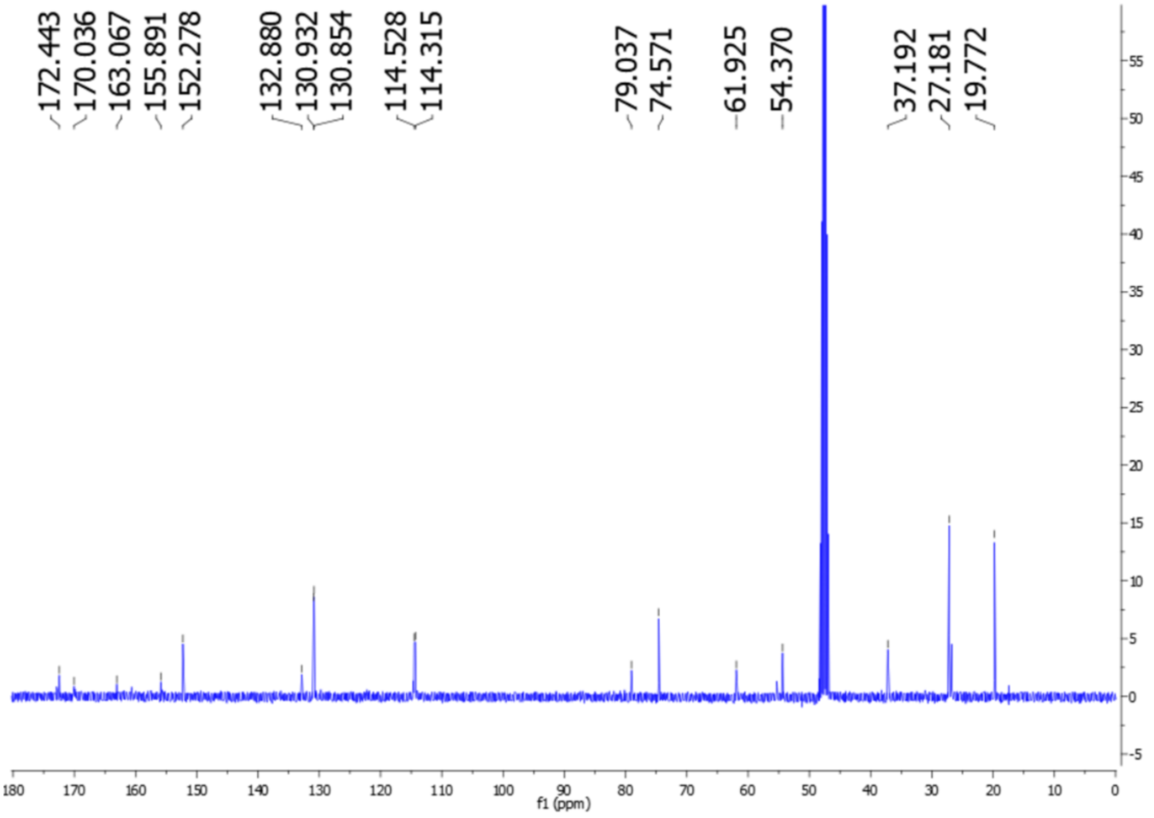

^{19}F NMR spectrum of compound **F1**, acquired in CD_3OD

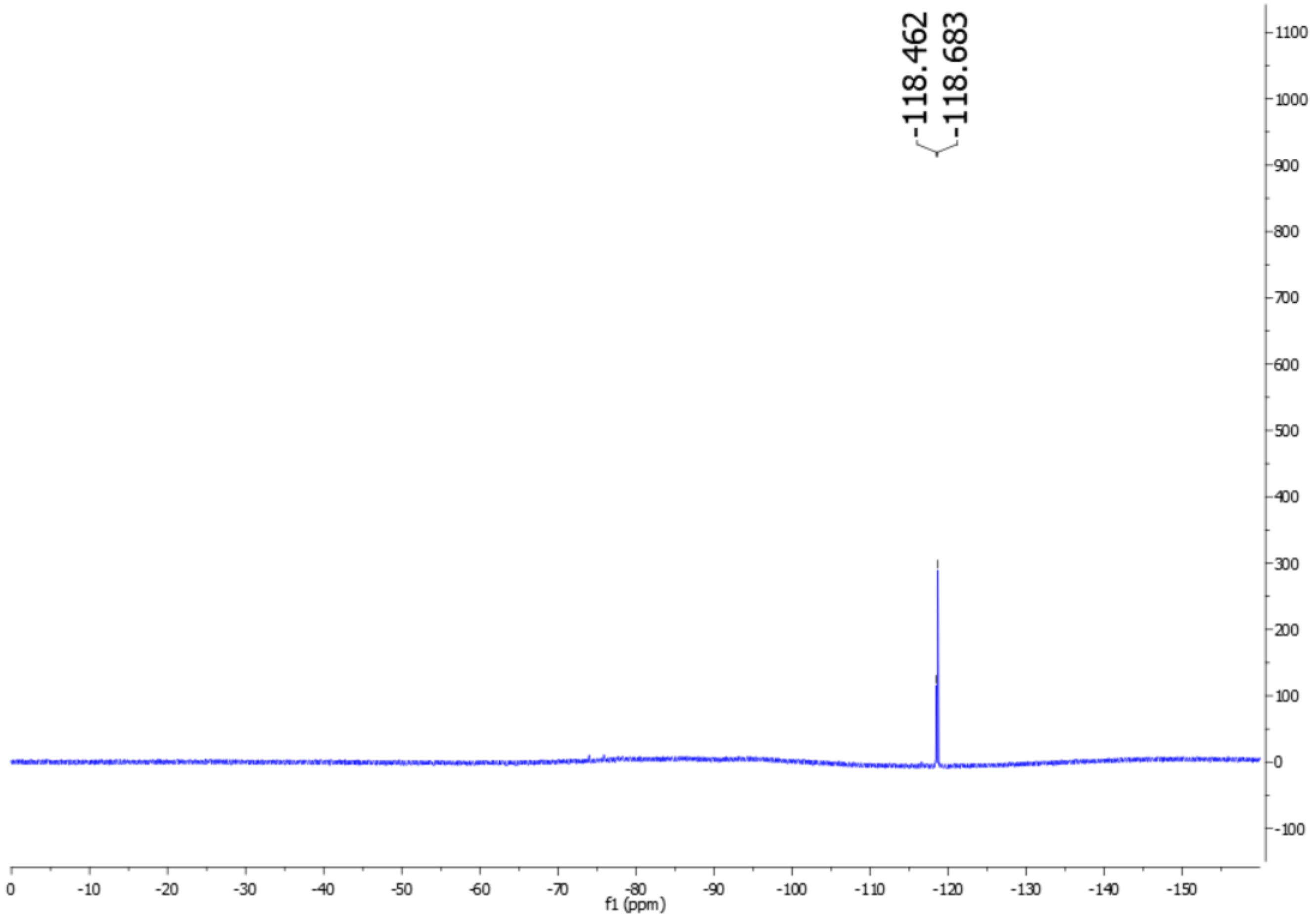

ATR-IR spectrum of compound **F1**

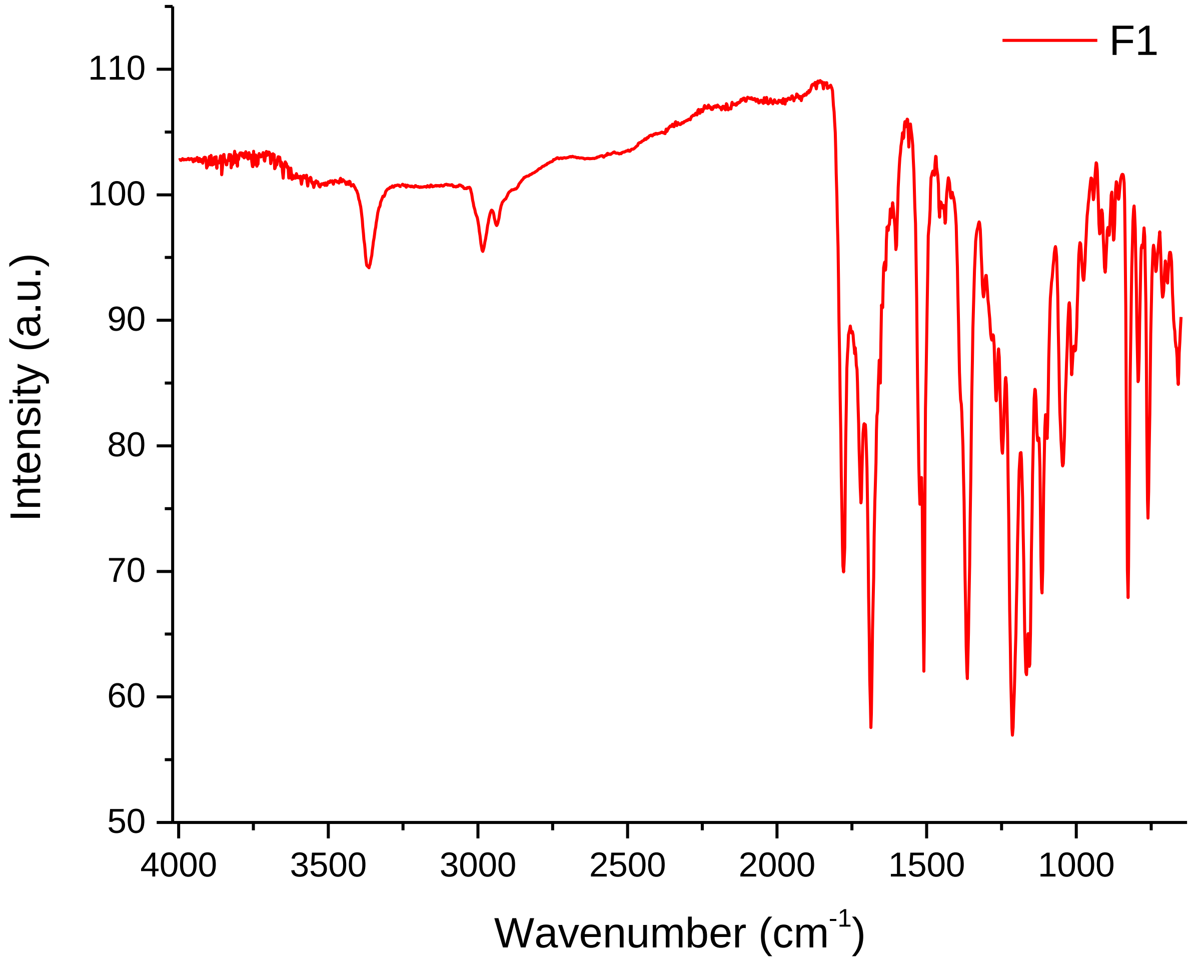

Table S1. Detailed list of the samples prepared with the solvent switch method

Entry	Conc.	Gelator	Solvent	Trigger	Outcome	Recovery
**1**	0.5%	**F0**	EtOH (30%)	Water (70%)	G	G
**2**	0.5%	**F0**	EtOH (50%)	Water (50%)	S	/
**3**	0.5%	**F0**	EtOH (70%)	Water (30%)	S	/
**4**	0.5%	**F1**	EtOH (30%)	Water (70%)	G	G
**5**	0.5%	**F1**	EtOH (50%)	Water (50%)	S	/
**6**	0.5%	**F1**	EtOH (70%)	Water (30%)	S	/
**7**	0.5%	**F2**	EtOH (30%)	Water (70%)	G	G
**8**	0.5%	**F2**	EtOH (50%)	Water (50%)	S	/
**9**	0.5%	**F2**	EtOH (70%)	Water (30%)	S	/
**10**	0.5%	**F0**	^{i}PrOH (30%)	Water (70%)	S	/
**11**	0.5%	**F0**	^{i}PrOH (50%)	Water (50%)	S	/
**12**	0.5%	**F0**	^{i}PrOH (70%)	Water (30%)	S	/
**13**	0.5%	**F1**	^{i}PrOH (30%)	Water (70%)	G	VS
**14**	0.5%	**F1**	^{i}PrOH (50%)	Water (50%)	S	/
**15**	0.5%	**F1**	^{i}PrOH (70%)	Water (30%)	S	/
**16**	0.5%	**F2**	^{i}PrOH (30%)	Water (70%)	G	VS
**17**	0.5%	**F2**	^{i}PrOH (50%)	Water (50%)	S	/
**18**	0.5%	**F2**	^{i}PrOH (70%)	Water (30%)	S	/
**19**	1.0%	**F0**	EtOH (30%)	Water (70%)	G	G
**20**	1.0%	**F0**	EtOH (50%)	Water (50%)	S	/
**21**	1.0%	**F0**	EtOH (70%)	Water (30%)	S	/
**22**	1.0%	**F1**	EtOH (30%)	Water (70%)	G	G
**23**	1.0%	**F1**	EtOH (50%)	Water (50%)	S	/
**24**	1.0%	**F1**	EtOH (70%)	Water (30%)	S	/
**25**	1.0%	**F2**	EtOH (30%)	Water (70%)	G	G
**26**	1.0%	**F2**	EtOH (50%)	Water (50%)	S	/
**27**	1.0%	**F2**	EtOH (70%)	Water (30%)	S	/
**28**	1.0%	**F0**	^{i}PrOH (30%)	Water (70%)	G	G
**29**	1.0%	**F0**	^{i}PrOH (50%)	Water (50%)	S	/
**30**	1.0%	**F0**	^{i}PrOH (70%)	Water (30%)	S	/
**31**	1.0%	**F1**	^{i}PrOH (30%)	Water (70%)	G	G
**32**	1.0%	**F1**	^{i}PrOH (50%)	Water (50%)	P	/
**33**	1.0%	**F1**	^{i}PrOH (70%)	Water (30%)	S	/
**34**	1.0%	**F2**	^{i}PrOH (30%)	Water (70%)	G	G
**35**	1.0%	**F2**	^{i}PrOH (50%)	Water (50%)	S	/
**36**	1.0%	**F2**	^{i}PrOH (70%)	Water (30%)	S	/

G = gel; S = solution; VS = viscous solution; P = precipitate

Table S2. Detailed list of the samples prepared with the pH change method and the addition of calcium chloride

Entry	Methodology	Conc.	Gelator	Solvent [a]	Trigger (eq)	Outcome	pH	Recovery
**37**	**b**	0.5%	**F0**	0.03 M PBS	GdL (1.4)	G	3.8	G
**38**	**b**	0.5%	**F1**	0.03 M PBS	GdL (1.4)	G	3.9	G
**39**	**b**	0.5%	**F2**	0.03 M PBS	GdL (1.4)	G	4.2	G
**40**	**c**	0.5%	**F0**	0.03 M PBS	$CaCl_2$ (1.0)	PG	/	/
**41**	**c**	0.5%	**F1**	0.03 M PBS	$CaCl_2$ (1.0)	G	4.4	PG
**42**	**c**	0.5%	**F2**	0.03 M PBS	$CaCl_2$ (1.0)	G	4.6	PG
**43**	**b**	1.0%	**F0**	0.06 M PBS	GdL (1.4)	G	3.6	G
**44**	**b**	1.0%	**F1**	0.06 M PBS	GdL (1.4)	G	3.7	G
**45**	**b**	1.0%	**F2**	0.06 M PBS	GdL (1.4)	G	3.8	G
**46**	**c**	1.0%	**F0**	0.06 M PBS	$CaCl_2$ (1.0)	G	4.5	G
**47**	**c**	1.0%	**F1**	0.06 M PBS	$CaCl_2$ (1.0)	G	4.7	G
**48**	**c**	1.0%	**F2**	0.06 M PBS	$CaCl_2$ (1.0)	G	4.8	G

[a] Final concentration; G = gel; PG = partial gel

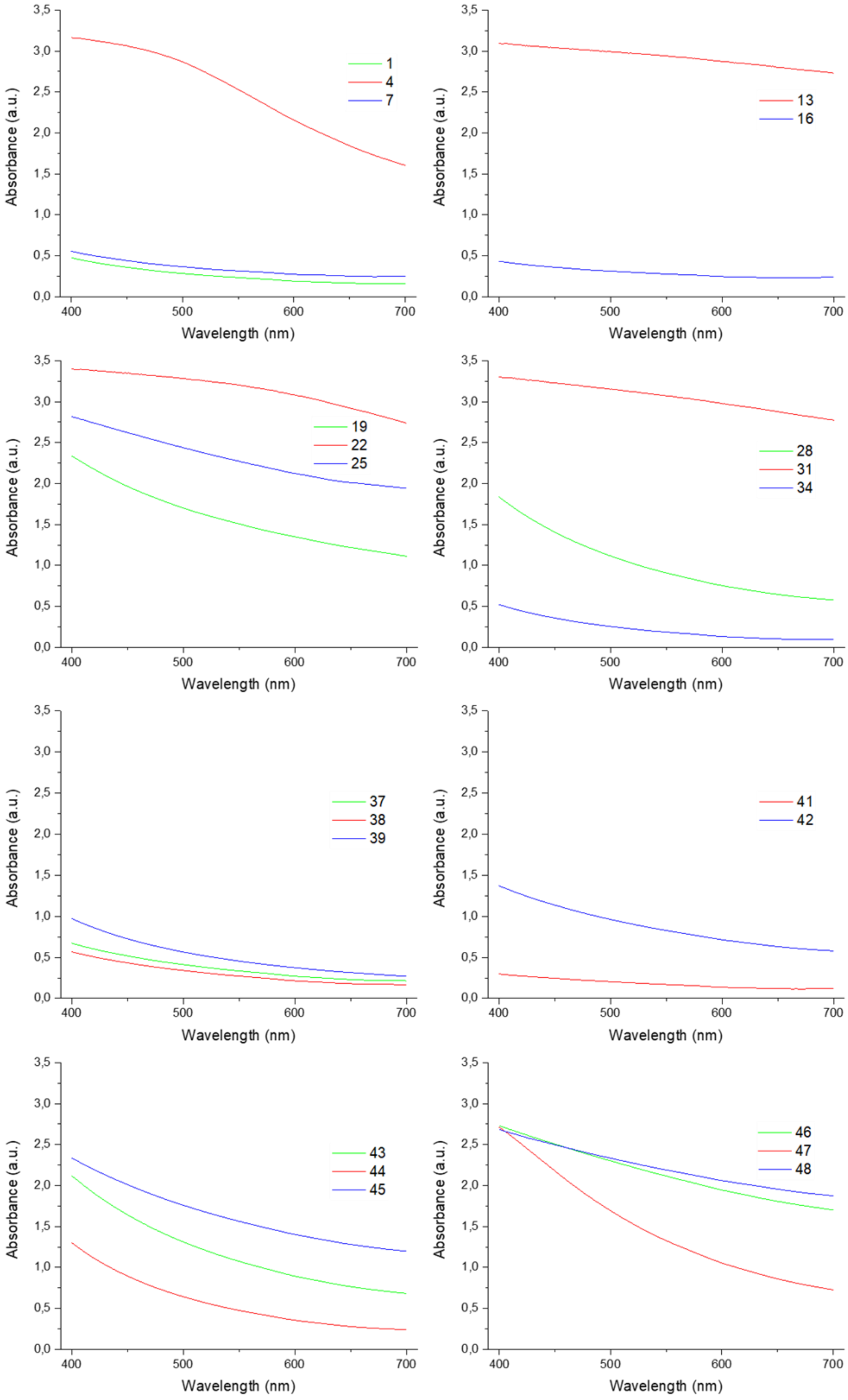

Figure S1. Absorbance spectra of the gels samples.

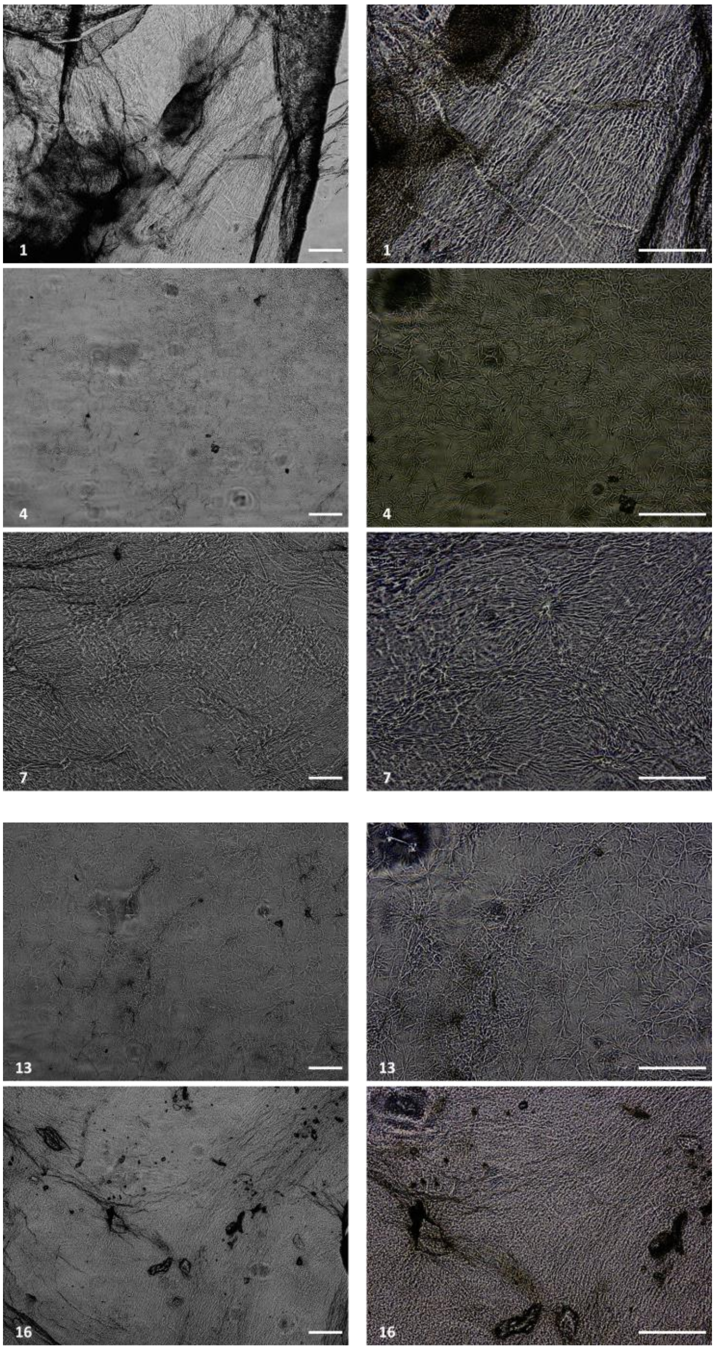

Figure S2. Morphology of the dried hydrogels **1, 4, 7, 13** and **16**, analysed through an optical microscope with different magnifications. Left: 10x magnification. Right: 20x magnification. For all the images the scalebar is 100 μm.

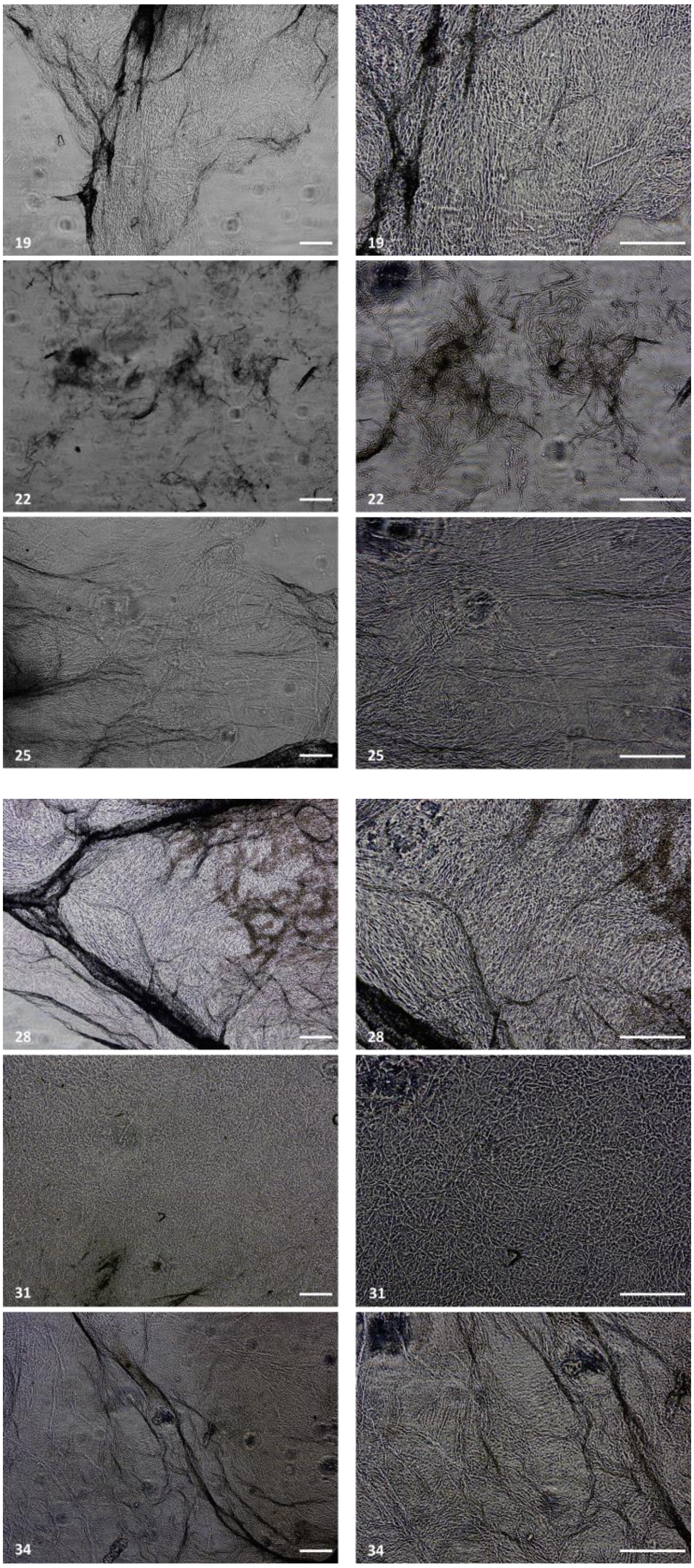

Figure S3. Morphology of the dried hydrogels **19, 22, 25, 28, 31** and **34**, analysed through an optical microscope with different magnifications. Left: 10x magnification. Right: 20x magnification. For all the images the scalebar is 100 μm.

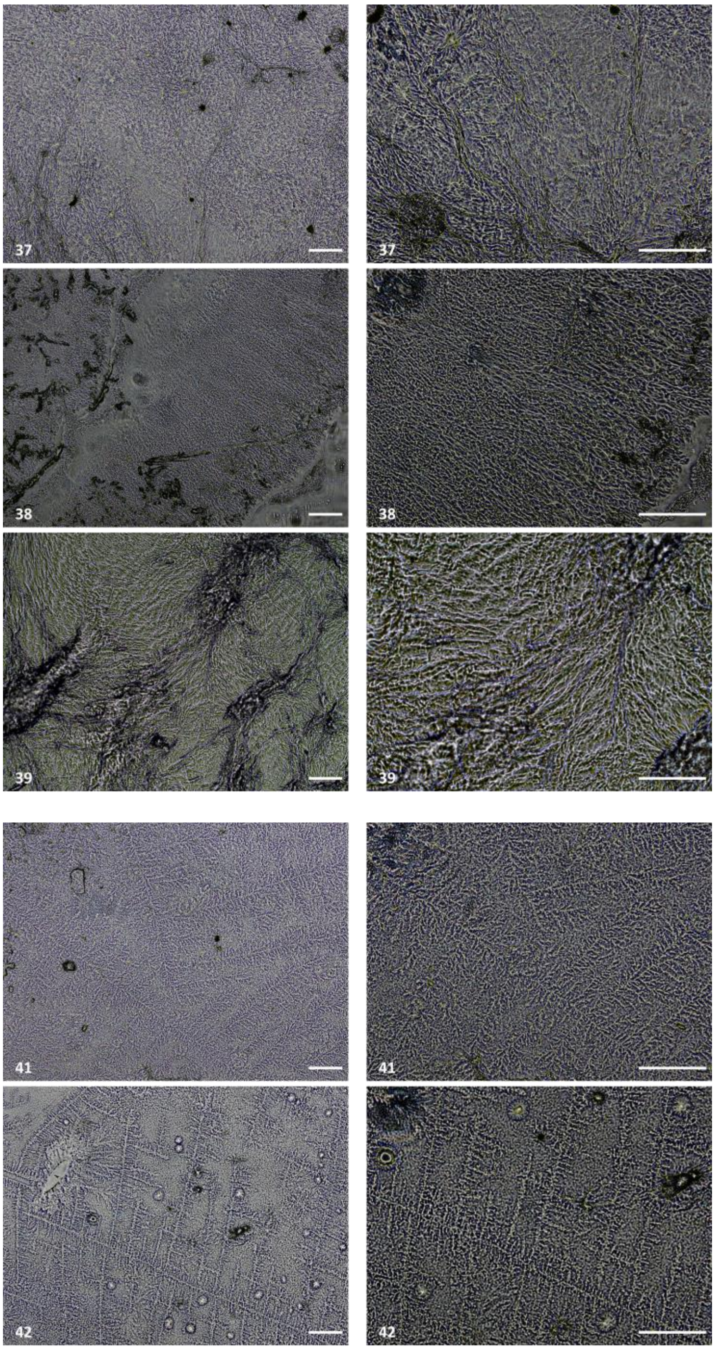

Figure S4. Morphology of the dried hydrogels **37, 38, 39, 41** and **42**, analysed through an optical microscope with different magnifications. Left: 10x magnification. Right: 20x magnification. For all the images the scalebar is 100 μm.

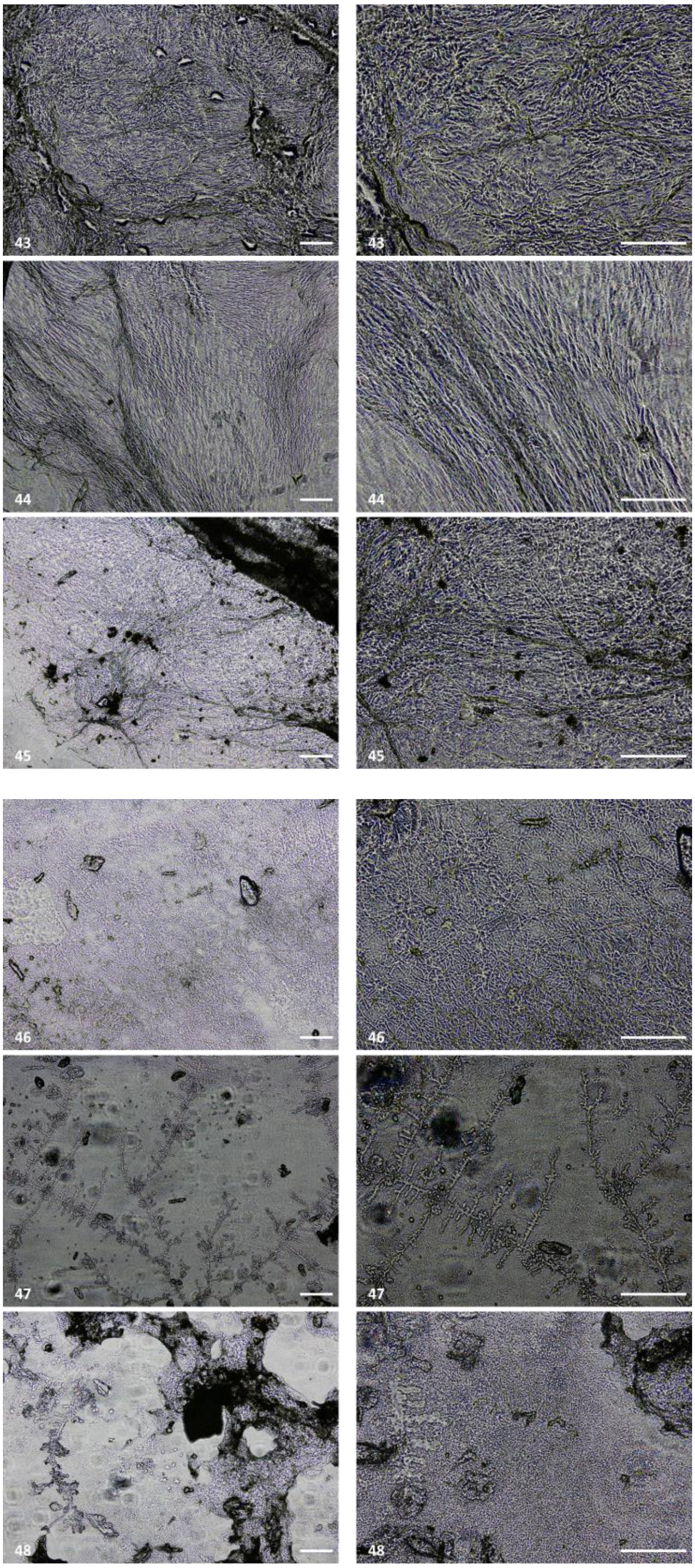

Figure S5. Morphology of the dried hydrogels **43, 44, 45, 46, 47** and **48**, analysed through an optical microscope with different magnifications. Left: 10x magnification. Right: 20x magnification. For all the images the scalebar is 100 μm.

Ethanol/water		
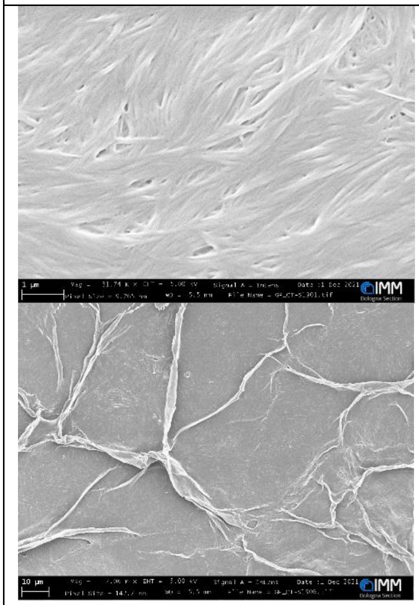<br>F0 – Sample 19	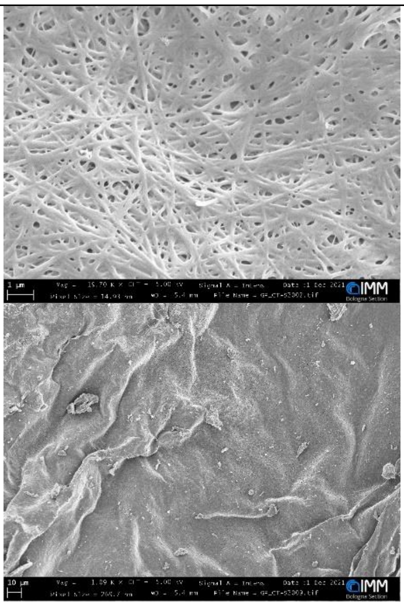<br>F1 – Sample 22	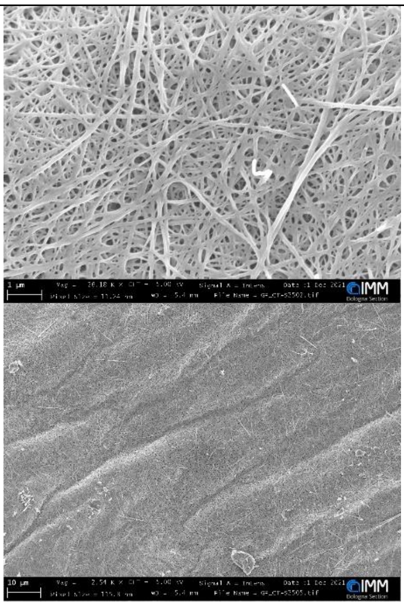<br>F2 – Sample 25
Isopropanol/water		
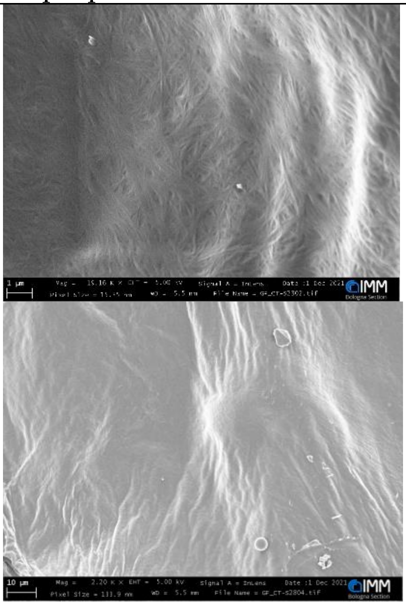<br>F0 – Sample 28	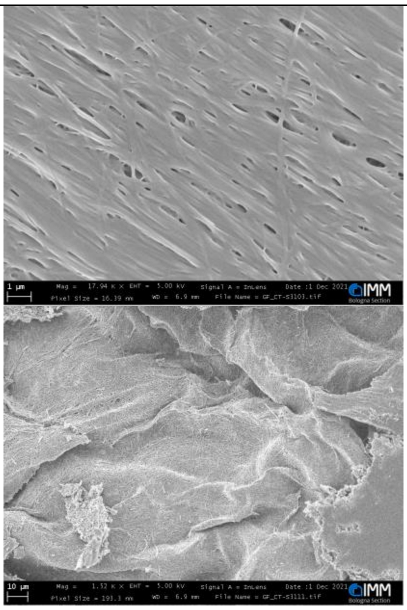<br>F1 – Sample 31	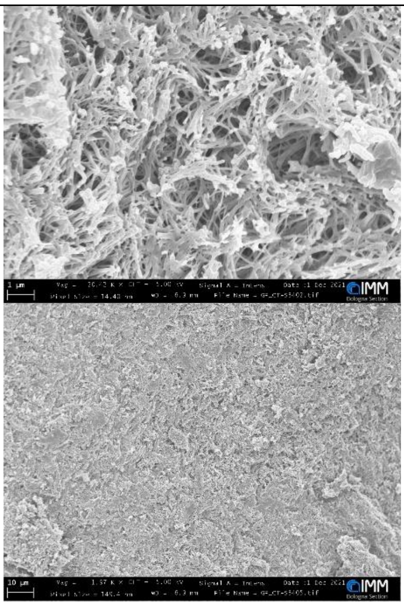<br>F2 – Sample 34

Figure S6. SEM images of the dried hydrogels **19, 22, 25, 28, 31** and **34**.

PBS/GdL		
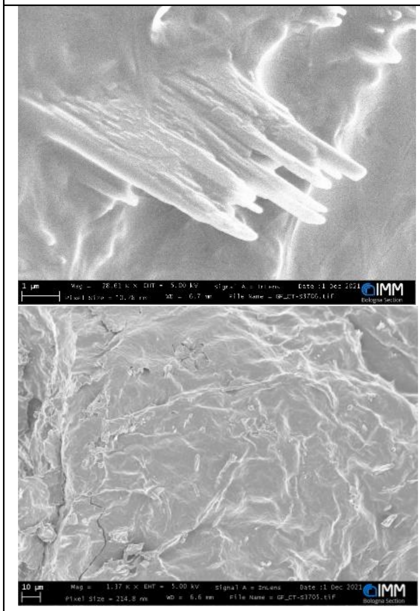	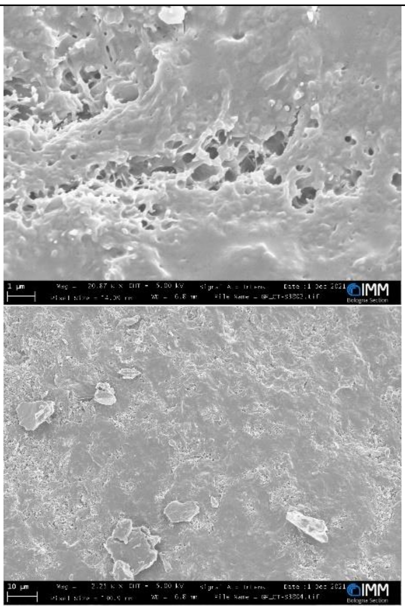	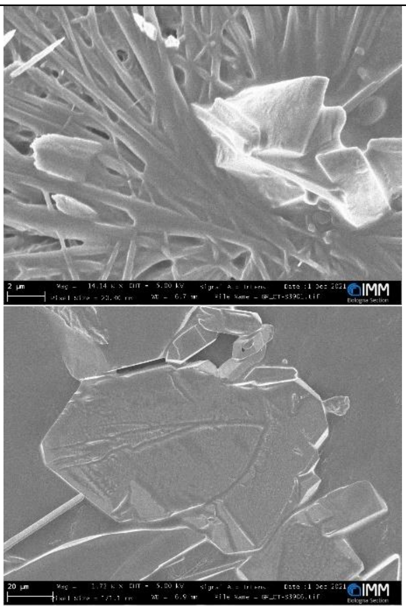
F0 - 43	F1 – Sample 44	F2 – Sample 45
PBS/$CaCl_2$		
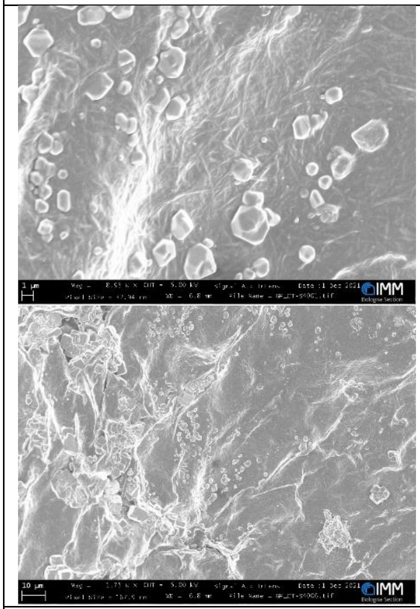	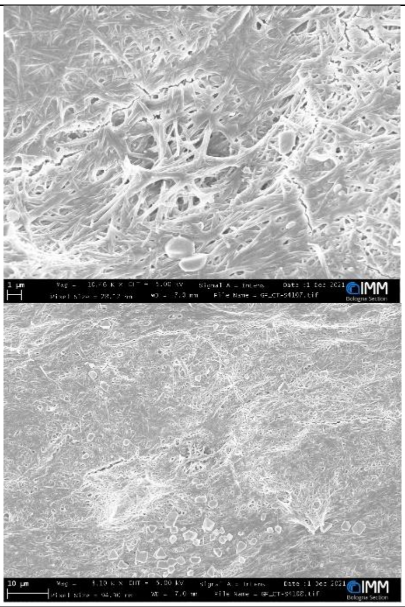	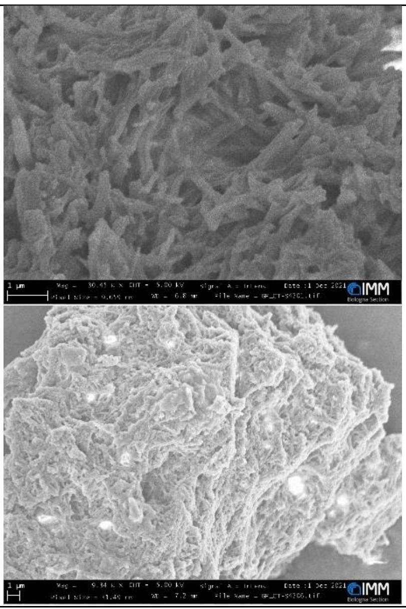
F0 – Sample 46	F1 – Sample 47	F2 – Sample 48

Figure S7. SEM images of the dried hydrogels **43-48**.

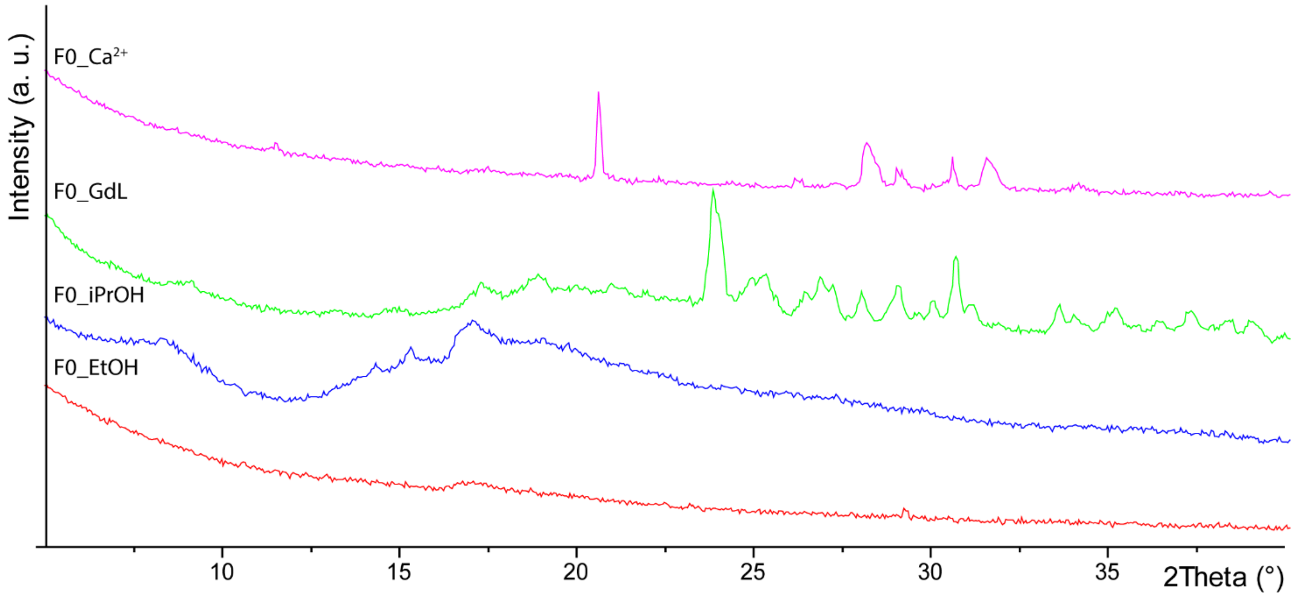

Figure S8. X-ray powder diffraction patterns of the xerogels from molecules F0 obtained from different chemical systems. F0_EtOH, F0_iPrOH, F0_GdL, and F0_Ca^{2+} correspond to **19**, **28**, **37** and **40**, respectively.

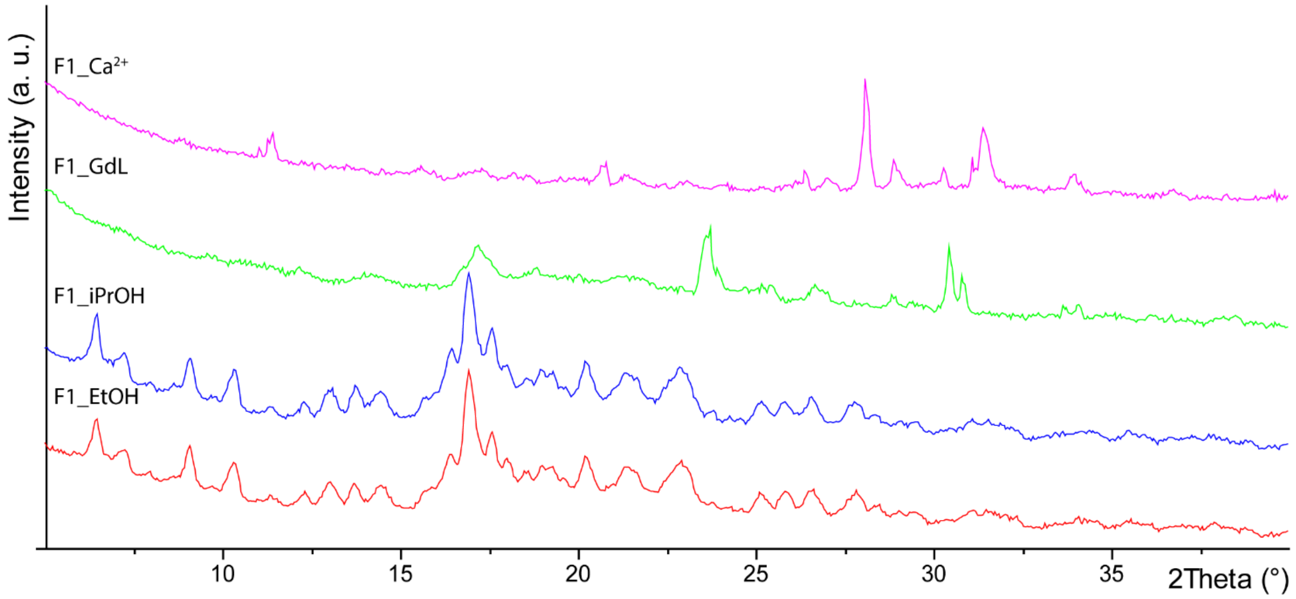

Figure S9. X-ray powder diffraction patterns of the xerogels from molecules F0 obtained from different chemical systems. F1_EtOH, F1_iPrOH, F1_GdL, and F1_Ca^{2+} correspond to **22**, **31**, **38** and **41**, respectively.

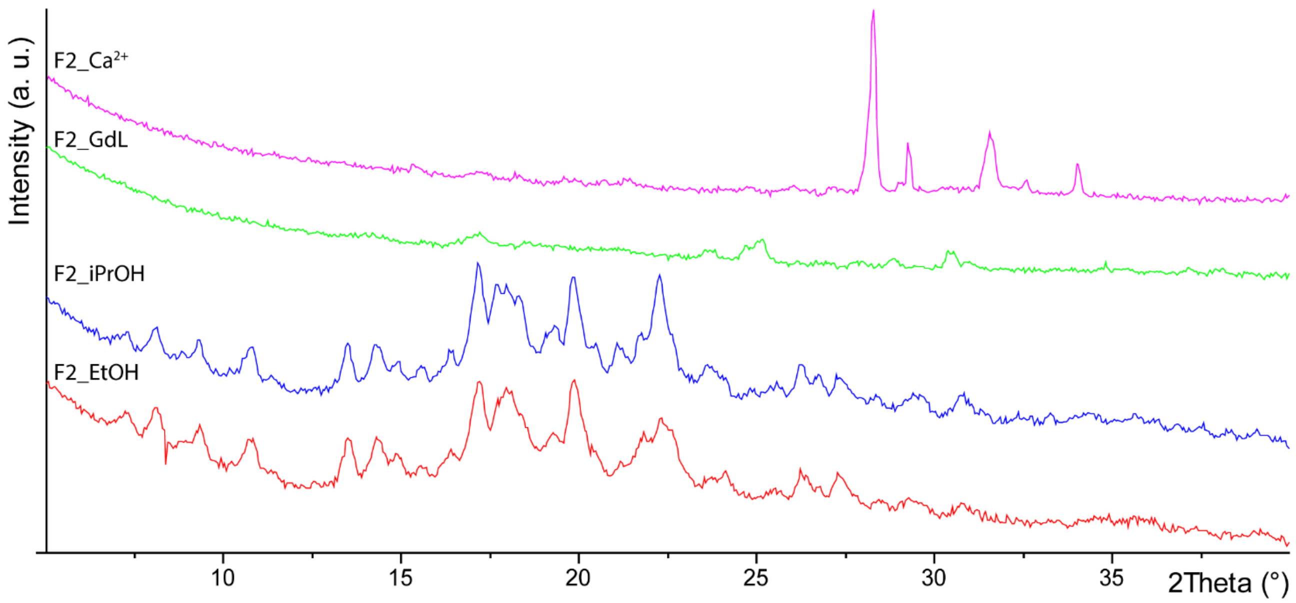

Figure S10. X-ray powder diffraction patterns of the xerogels from molecules F0 obtained from different chemical systems. F2_EtOH, F2_iPrOH, F2_GdL, and F2_Ca^{2+} correspond to **25**, **34**, **39** and **42**, respectively.

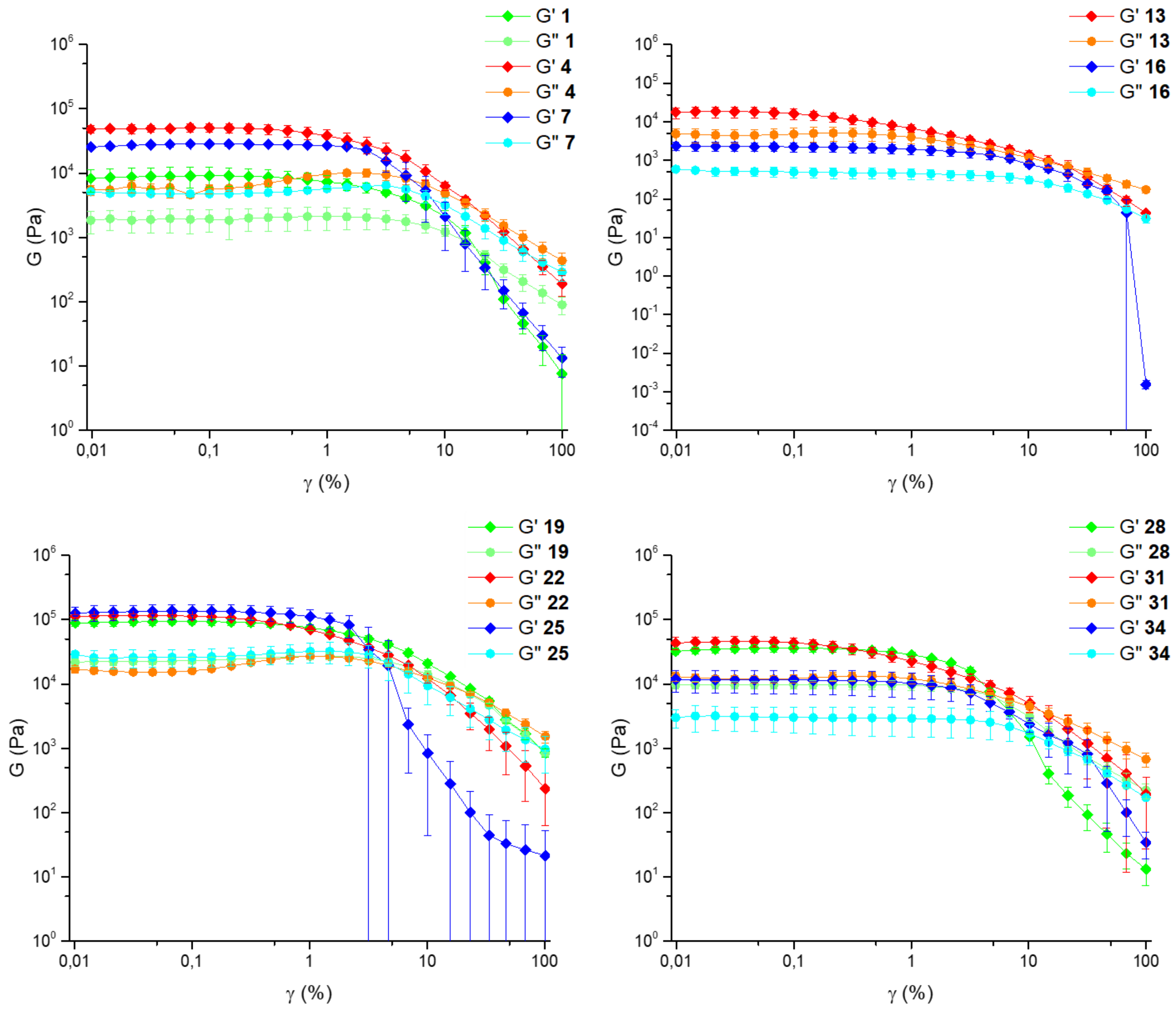

Figure S11. Amplitude sweep analysis of the samples **1, 4, 7, 13, 16, 19, 22, 25, 28, 31** and **34.**

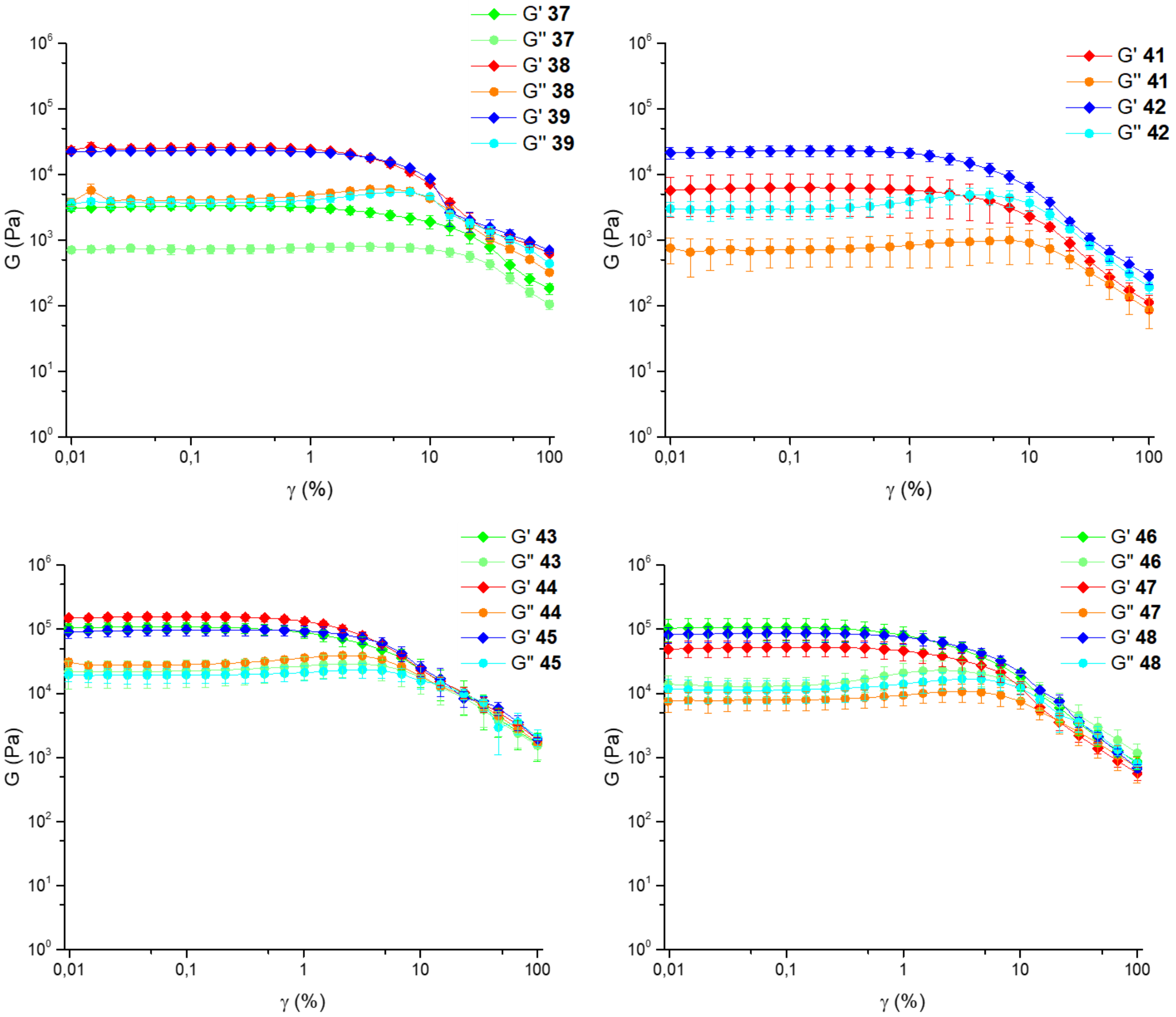

Figure S12. Amplitude sweep analysis of the samples **37, 38, 39** and **41-48**.

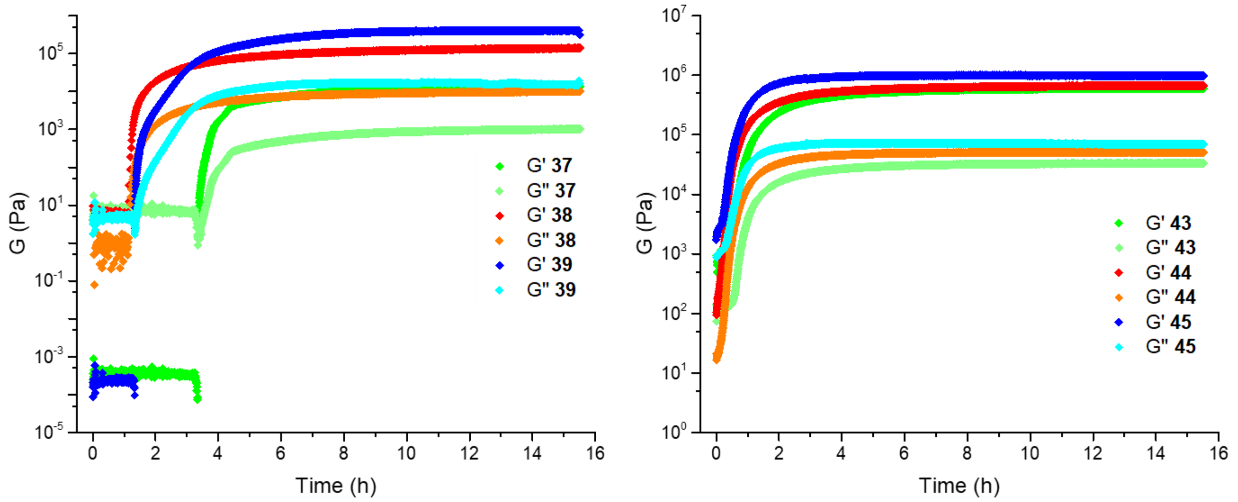

Figure S13. Time sweep analysis of the samples **37-39** and **43-45** obtained with the addition of GdL.

Table S3. Summary of the Properties of Gels

Sample	Gelator	Conc.	Recovery	pH	A (λ=630 nm)	T (%)	G′ (kPa) (γ = 0.068%)	G″ (kPa) (γ = 0.068%)
EtOH/H_2O								
**1**	**F0**	0.5	yes	n.a.	0.17469	**66.88**	9.06 ± 3.27	1.91 ± 0.77
**4**	**F1**	0.5	yes	n.a.	1.96473	1.08	51.18 ± 8.04	4.59 ± 0.54
**7**	**F2**	0.5	yes	n.a.	**0.26185**	**54.72**	**28.29 ± 2.47**	**4.74 ± 0.32**
**19**	**F0**	1.0	yes	n.a.	1.26706	5.41	93.79 ± 11.23	22.80 ± 2.73
**22**	**F1**	1.0	yes	n.a.	2.98876	0.10	114.96 ± 6.24	15.44 ± 1.17
**25**	**F2**	1.0	yes	n.a.	2.04926	0.89	135.93 ± 35.36	25.94 ± 7.91
iPrOH/H_2O								
**13**	**F1**	0.5	no	n.a.	2.83436	0.15	17.26 ± 5.43	4.57 ± 1.79
**16**	**F2**	0.5	no	n.a.	0.23566	**58.12**	2.25 ± 0.58	0.51 ± 0.15
**28**	**F0**	1.0	yes	n.a.	0.68608	20.60	36.07 ± 4.62	9.74 ± 1.36
**31**	**F1**	1.0	yes	n.a.	2.92284	0.12	45.29 ± 8.55	11.93 ± 1.71
**34**	**F2**	1.0	yes	n.a.	0.11273	**77.14**	11.61 ± 4.58	3.06 ± 1.30
PBS/GdL								
**37**	**F0**	0.5	yes	3.8	0.24547	56.82	3.29 ± 0.35	0.73 ± 0.12
**38**	**F1**	0.5	yes	3.9	**0.19197**	**64.27**	**25.39 ± 3.00**	**4.06 ± 0.42**
**39**	**F2**	0.5	yes	4.2	**0.33574**	**46.16**	**23.15 ± 1.31**	**3.73 ± 0.57**
**43**	**F0**	1.0	yes	3.6	0.8128	15.39	109.16 ± 18.09	22.21 ± 10.22
**44**	**F1**	1.0	yes	3.7	0.30614	49.41	157.02 ± 18.62	27.78 ± 2.03
**45**	**F2**	1.0	yes	3.8	1.32719	4.71	97.00 ± 18.39	19.30 ± 5.16
PBS/Ca^{2+}								
**41**	**F1**	0.5	no	4.4	0.12512	**74.97**	6.23 ± 3.93	0.71 ± 0.33
**42**	**F2**	0.5	no	4.6	0.66275	21.74	22.85 ± 4.27	2.94 ± 0.91
**46**	**F0**	1.0	yes	4.5	1.86026	1.38	106.98 ± 41.32	12.90 ± 4.31
**47**	**F1**	1.0	yes	4.7	0.93284	11.67	52.18 ± 14.65	7.83 ± 2.54
**48**	**F2**	1.0	yes	4.8	1.99850	1.00	86.82 ± 23.94	11.13 ± 4.39

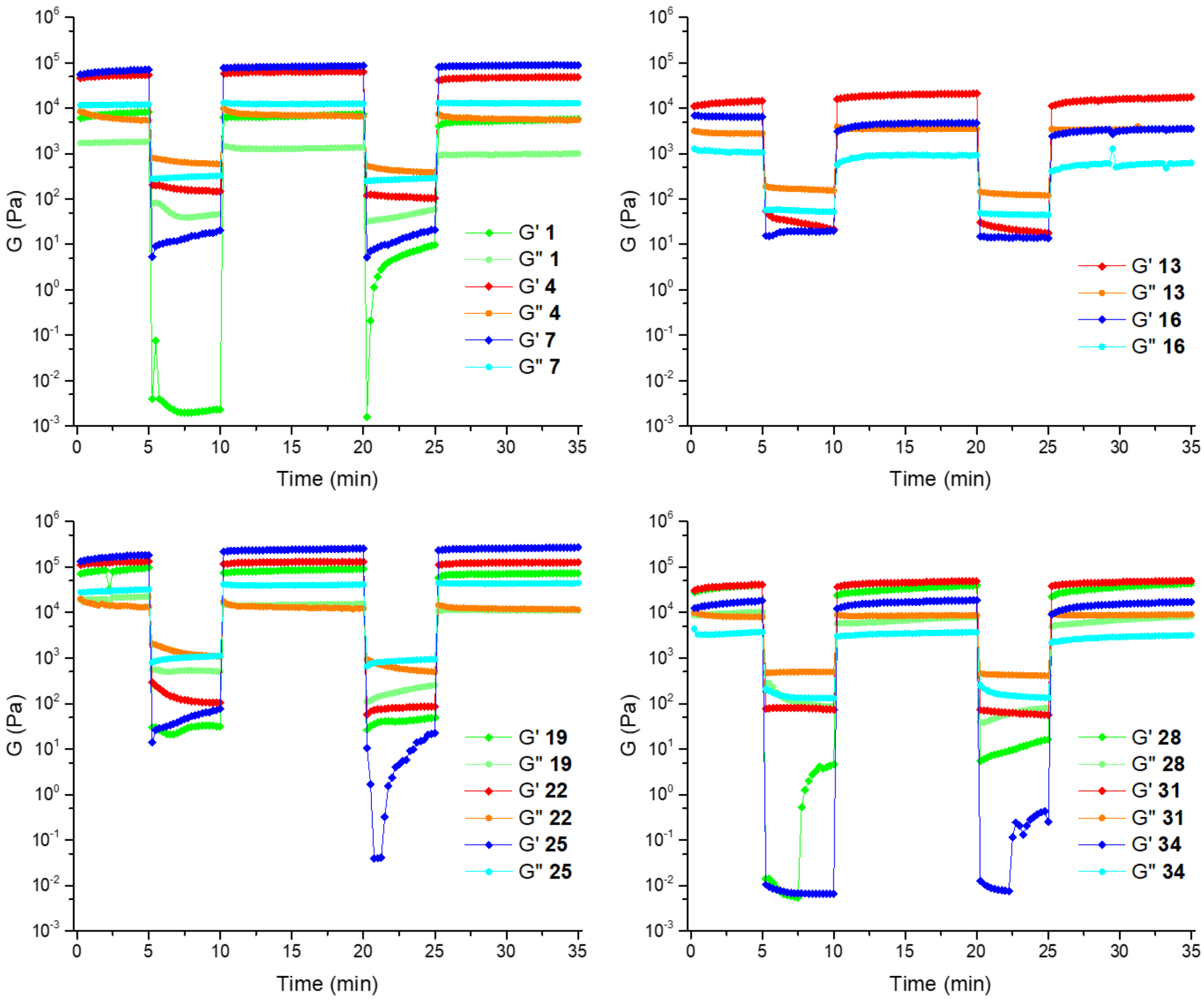

Figure S14. Thixotropic behaviour of the samples **1, 4, 7, 13, 16, 19, 22, 25, 28, 31** and **34** analysed with the rheometer.

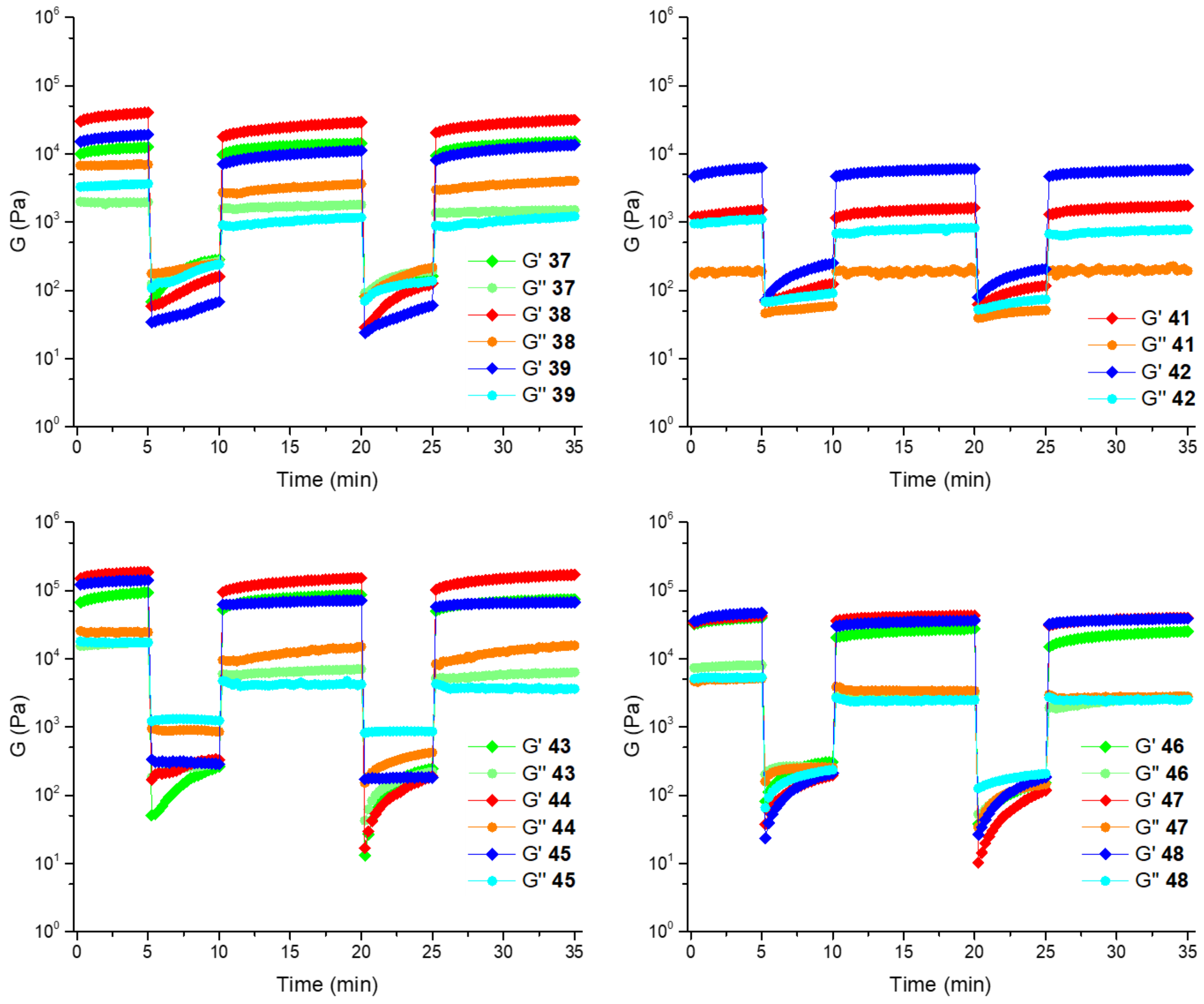

Figure S15. Thixotropic behaviour of the samples **37, 38, 39** and **41**-**48**, analysed with the rheometer.

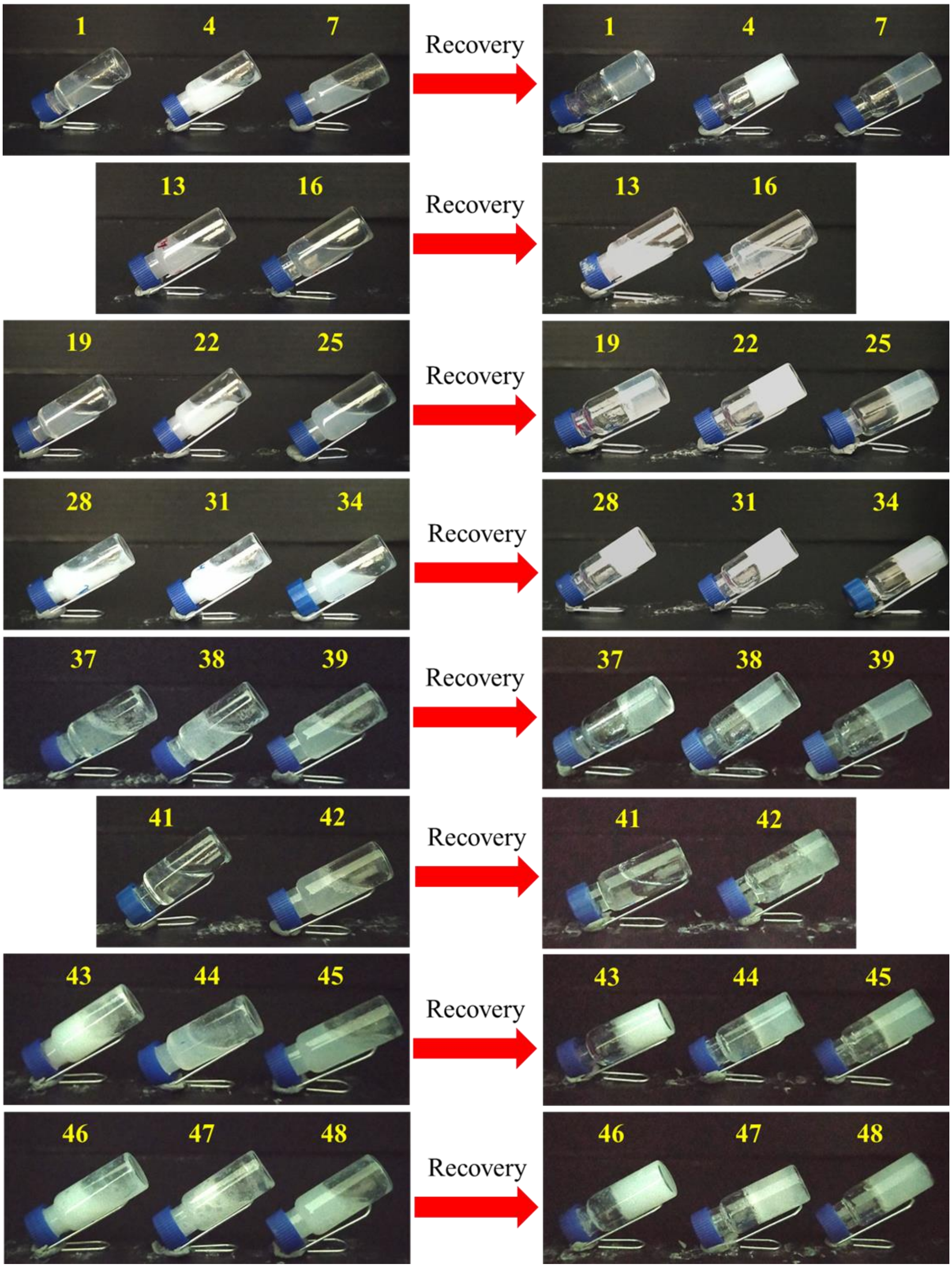

Figure S16. Thixotropic behaviour of the gels formed after vigorous shaking (left) and 16 hours of recovery (right).

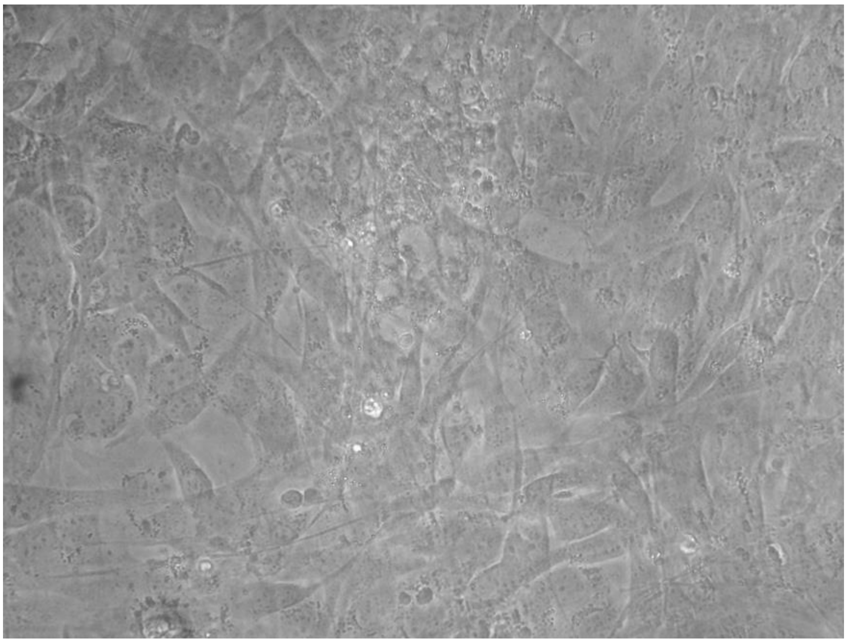

Figure S17. Optical micrograph of cells in DPBS (control).
